# Identification of drivers of mycobacterial resistance to peptidoglycan synthesis inhibitors

**DOI:** 10.3389/fmicb.2022.985871

**Published:** 2022-09-06

**Authors:** Francisco Olivença, Cláudia Ferreira, Alexandra Nunes, Cátia Silveiro, Madalena Pimentel, João Paulo Gomes, Maria João Catalão

**Affiliations:** ^1^Faculty of Pharmacy, Research Institute for Medicines (iMed.ULisboa), Universidade de Lisboa, Lisbon, Portugal; ^2^Genomics and Bioinformatics Unit, Department of Infectious Diseases, National Institute of Health, Lisbon, Portugal; ^3^Faculty of Veterinary Medicine, Lusófona University, Lisbon, Portugal

**Keywords:** mycobacteria, tuberculosis, antimicrobial resistance, beta-lactams, beta-lactamase, WGS

## Abstract

Beta-lactams have been excluded from tuberculosis therapy due to the intrinsic resistance of *Mycobacterium tuberculosis* (*Mtb*) to this antibiotic class, usually attributed to a potent beta-lactamase, BlaC, and to an unusually complex cell wall. In this pathogen, the peptidoglycan is cross-linked by penicillin-binding proteins (PBPs) and L,D-transpeptidases, the latter resistant to inhibition by most beta-lactams. However, recent studies have shown encouraging results of beta-lactam/beta-lactamase inhibitor combinations in clinical strains. Additional research on the mechanisms of action and resistance to these antibiotics and other inhibitors of peptidoglycan synthesis, such as the glycopeptides, is crucial to ascertain their place in alternative regimens against drug-resistant strains. Within this scope, we applied selective pressure to generate mutants resistant to amoxicillin, meropenem or vancomycin in *Mtb* H37Rv or *Mycolicibacterium smegmatis* (*Msm*) mc^2^-155. These were phenotypically characterized, and whole-genome sequencing was performed. Mutations in promising targets or orthologue genes were inspected in *Mtb* clinical strains to establish potential associations between altered susceptibility to beta-lactams and the presence of key genomic signatures. The obtained isolates had substantial increases in the minimum inhibitory concentration of the selection antibiotic, and beta-lactam cross-resistance was detected in *Mtb*. Mutations in L,D-transpeptidases and major PBPs, canonical targets, or BlaC were not found. The transcriptional regulator PhoP (Rv0757) emerged as a common denominator for *Mtb* resistance to both amoxicillin and meropenem, while Rv2864c, a lipoprotein with PBP activity, appears to be specifically involved in decreased susceptibility to the carbapenem. Nonetheless, the mutational pattern detected in meropenem-resistant mutants was different from the yielded by amoxicillin-or vancomycin-selected isolates, suggesting that distinct pathways may participate in increased resistance to peptidoglycan inhibitors, including at the level of beta-lactam subclasses. Cross-resistance between beta-lactams and antimycobacterials was mostly unnoticed, and *Msm* meropenem-resistant mutants from parental strains with previous resistance to isoniazid or ethambutol were isolated at a lower frequency. Although cell-associated nitrocefin hydrolysis was increased in some of the isolates, our findings suggest that traditional assumptions of *Mtb* resistance relying largely in beta-lactamase activity and impaired access of hydrophilic molecules through lipid-rich outer layers should be challenged. Moreover, the therapeutical potential of the identified *Mtb* targets should be explored.

## Introduction

Beta-lactams are a successful antibiotic class, with worldwide accessibility, a relatively safe adverse events profile, and a broad action spectrum against both Gram-negative and Gram-positive bacteria. Since their discovery, beta-lactams have been extensively used in the treatment of multiple infectious diseases, ultimately leading to development of resistance in several species. Four major mechanisms of resistance to beta-lactams are described: i) hydrolysis of the antibiotic due to beta-lactamase activity; ii) blocking of the access of the antibiotic into the cell by reduced cell wall (CW) permeability; iii) extrusion through efflux pumps, which prevents its concentration within the cell; iv) and modifications of the target enzymes, usually causing reduction or loss of drug affinity (Drawz and Bonomo, 2010; Fisher and Mobashery, 2016).

Despite their favorable characteristics, beta-lactam antibiotics have not been systematically applied in the treatment of tuberculosis (TB) due to the intrinsic resistance of *Mycobacterium tuberculosis* (*Mtb*) to this class, mostly credited to the potent BlaC, a chromosomally encoded and highly effective beta-lactamase (Flores et al., 2005). Beta-lactam resistance in this pathogen is additionally associated with the complex and highly hydrophobic outer layer of the CW of *Mtb,* which creates an impermeable barrier for hydrophilic compounds (Jankute et al., 2015), and to the presence of non-canonical peptidoglycan (PG) crosslinks. D,D-transpeptidases, also known as penicillin-binding proteins (PBPs), are responsible for the 4 → 3 crosslinking of neighboring stem peptides in most bacteria. Significantly, mycobacteria additionally possess L,D-transpeptidases (Ldts), that in coordination with D,D-carboxypeptidases catalyze the unusual 3 → 3 crosslinks (Cordillot et al., 2013; Baranowski et al., 2018). *Mtb* and *Mycolicibacterium smegmatis* (*Msm*), respectively, have five and six known Ldts, and both have several enzymes associated with D,D-transpeptidase or D,D-carboxypeptidase activity (Patru and Pavelka, 2010; Kumar et al., 2012; Sanders et al., 2014; Tolufashe et al., 2020; Levine and Beatty, 2021). In contrast to PBPs, most Ldts are inactivated only by carbapenems, a subgroup of beta-lactams (Cordillot et al., 2013). Vancomycin, a glycopeptide that inhibits synthesis of PG through a distinct action mechanism from the one employed by beta-lactams, is also considered ineffective in *Mtb*. Resistance to this antibiotic is poorly understood, but increased susceptibility to the glycopeptide and amoxicillin/clavulanate was associated with the absence of two major Ldts in *Mtb* (Schoonmaker et al., 2014).

The development of the potent subclass of carbapenems and the addition of clavulanate or other protective beta-lactamase inhibitors (Hugonnet and Blanchard, 2007), have led to the revaluation of the role of the beta-lactams within TB regimens (Story-Roller and Lamichhane, 2018; Catalão et al., 2019). Results from both *in vitro* and *in vivo* studies support the potential of carbapenems as anti-TB drugs (Hugonnet et al., 2009; England et al., 2012; Solapure et al., 2013; Zhang et al., 2016; Kaushik et al., 2017; Olivença et al., 2022), and several phase 2 clinical trials (NCT02349841, NCT02381470 and NCT03174184) have been recently undertaken to determine early bactericidal activity of regimens containing beta-lactams.

Given the emergence of drug-resistant strains and the necessity to design alternative schemes, WHO currently places two carbapenems in group C of the drugs recommended for use in longer multidrug-resistant TB (MDR-TB) treatments. Due to their effectiveness in the evidence reviews, the agency considers that additional research on the role carbapenems may play in MDR-TB regimens is important [World Health Organization (WHO), 2020].

The introduction of a new antibiotic to a treatment regimen carries the risk of resistance development. Thus, to ensure a safe and optimized application, it is essential to characterize its respective resistance-inducing potential and the underlining mechanisms. This is particularly crucial for beta-lactams since few studies on the determinants of resistance to these antibiotics in *Mtb* are available (Cohen et al., 2016; Xu et al., 2017; Olivença et al., 2022). Associations between altered beta-lactam susceptibility of clinical strains and the presence of individual mutations in genes encoding BlaC, major PBPs, Ldts, or D,D-carboxypeptidases appear to be infrequent (Cohen et al., 2016; Li et al., 2018; Olivença et al., 2022). Together with these observations, findings that lesser-known PBPs (Kumar et al., 2022) and redox balance (Mishra et al., 2017) may also contribute to resistance to beta-lactams, suggest that the reality is far more complex than formerly assumed.

A valid strategy to identify drivers of resistance consists of the isolation and characterization of spontaneous resistant mutants, followed by whole-genome sequencing (WGS) (O’Malley and Melief, 2015). This approach has been previously employed in *Mtb* to determine that bedaquiline-resistant mutants acquire mutations in the *atpE* and *Rv0678* genes (Degiacomi et al., 2020). The main purposes of this work are to expose the mechanisms and possible target genes involved in high-level beta-lactam resistance and to verify if there is cross-resistance between this class and other CW targeting antibiotics, such as isoniazid, ethambutol, or vancomycin. To achieve this, we generated a collection of *Mtb* and *Msm* drug-resistant mutants and characterized their phenotypes through minimum inhibitory concentration (MIC), nitrocefin hydrolysis, and ethidium bromide (EtBr) accumulation and efflux assays. We performed WGS to reveal chromosomal mutations putatively responsible for the phenotypes. The obtained results showed that both *Mtb* and *Msm* mutants displayed high resistance to beta-lactams, and that although some increase in beta-lactamase activity may be responsible for this, additional processes are probably at work. Moreover, potentially distinct pathways are implicated in resistance to amoxicillin and meropenem. Our outputs provide evidence on eventual therapeutic targets to enhance the activity of PG synthesis inhibitors and emphasize the need for a better clarification of the currently accepted paradigms of beta-lactam activity and resistance in *Mtb*.

## Materials and methods

### Bacterial strains, culture conditions and antibiotics

*Mtb* H37Rv, *Msm* mc^2^-155 and *Msm* PM965 (*ept-1 rpsL4 ΔblaS1*), a mutant strain deficient for the major beta-lactamase BlaS (Raymond et al., 2005), were used in this study. Bacteria were grown in Middlebrook 7H9 medium (BD Biosciences) or in Middlebrook 7H10 medium (BD Biosciences), supplemented with 0.2% or 0.5% of glycerol, respectively. Both media were supplemented with 10% oleic acid-albumin-dextrose-catalase (BD Difco) for *Mtb* and 0.5% glucose for *Msm*. Tyloxapol (Sigma-Aldrich) was added to liquid medium to a final concentration of 0.05% to prevent clump formation. Stocks of amoxicillin (AMX), biapenem (BIA), cefotaxime (CTX), doripenem (DOR), ertapenem (ETP), ethambutol (EMB), meropenem (MEM) and vancomycin (VAN) (Sigma-Aldrich) were prepared in purified water. Potassium clavulanate (CLA) (Sigma-Aldrich) was prepared in phosphate buffer pH 6.0, 0.1 M. Isoniazid (INH) and rifampicin (RIF) (Sigma-Aldrich) were prepared in dimethyl sulfoxide or methanol, respectively.

### Isolation of resistant mutants

Drug-resistant *Mtb* or *Msm* mutants were selected as described previously (O’Malley and Melief, 2015). Briefly, 10^7^, 10^8^ and 10^9^ CFU from exponentially growing cultures were plated onto agar plates supplemented with antibiotics at a concentration 5X, 10X, 20X, or 50X the MIC, previously determined in triplicates by the agar dilution method, following a protocol from Sirgel et al. (2009). The plates were incubated at 37°C until colonies were visible and countable, generally ranging between 4 to 6 weeks in *Mtb* and 3 to 7 days in *Msm*. Whenever possible, resistance frequency was calculated as the ratio of the number of colonies growing on antibiotic-containing plates to the total number of CFUs obtained on drug-free plates. Several colonies were selected from the plate with highest antibiotic concentration and lowest input inoculum and streaked on agar plates containing an antibiotic concentration equal to the one used for mutant isolation for confirmation of resistance in solid medium. Individual colonies were then picked and inoculated into liquid media. A schematic representation of the process applied to obtain the resistant mutants is summarized in Supplementary Figure S1.

### MIC determination assays

An adaptation of a broth microdilution assay was used to determine antibiotic MICs of the isolates (Cohen et al., 2016). Briefly, 10^5^ CFU/ml of log-phase cultures were added to 96-well plates containing serial dilutions of the antibiotics or drug-free medium. A negative control without bacterial inoculum was also included. Plates inoculated with *Mtb* or *Msm* were incubated at 37°C for 10–12 days or 2 days, respectively. MIC values were annotated as the lowest concentration of antibiotic without visible bacterial growth. The assay was performed three times for each isolate and parental strain, and median values were determined. Fold changes were calculated for each antibiotic as the log_2_ ratio of the MIC of each isolate to the MIC of the respective WT strain.

### Growth curves and spot assay

*Mtb* H37Rv wild type (WT) and resistant mutants were grown in 7H9 media to an OD_600_ of 0.5–0.6 and reinoculated into fresh medium to a normalized OD_600_ of 0.06. Flasks were incubated at 37°C without shaking for 15 days and growth curves were plotted by monitoring the OD_600_ of the cultures after 1, 2, 4, 7, 8, 11 and 15 days of incubation. Mean and standard deviation error values were calculated from assays with two biological replicates prepared for each isolate.

For the spot assays, exponential growth phase cultures were standardized to an OD_600_ of 0.25. These were then diluted 1:10 and 5 μl of each suspension were spotted onto antibiotic-containing or antibiotic-free plates. For each antibiotic, agar plates contained the same concentration employed in the isolates’ selection. The plates were incubated at 37°C until spots were visible.

### Nitrocefin hydrolysis assays

Beta-lactamase activity was accessed through hydrolysis of nitrocefin, a chromogenic cephalosporin (Flores et al., 2005). *Mtb* and *Msm* cultures were grown at 37°C until saturation (OD600 ≥ 1) and 5 ml were centrifuged at 3,000 *g* for 5 min. The supernatants were collected and placed on ice and the pellets were resuspended in 5 ml of ice-cold PBS and centrifuged again. The supernatants were discarded, and the cell pellets resuspended in 1 mL of a PBS solution containing protease inhibitor 1x (Calbiochem) and 10 μg/mL DNase I (NZYTech). Cells were disrupted in a Beadbug (Benchmark Scientific) by 3 cycles at maximum speed for 30 s, interspersed by 1 min resting periods on ice. The samples were then centrifuged at 10,000 *g* for 5 min at 4°C, and the supernatants were labeled as cell lysates. Both culture supernatants and cell lysate samples were filtered through 0.2 μm filters and kept on ice. Total protein concentrations were determined by Bradford method quantification and obtained from linear regression of a bovine serum albumin calibration curve.

Different volumes of the samples were transferred to a clear 96-well plate. PBS was added for a final total volume of 200 μL and 20 μL of nitrocefin were added to sample and control wells to a final concentration of 100 μM. Absorbance for *Mtb* and *Msm* samples was measured at 486 nm, every 30 s for a period of 15 min, in an Infinite M200 Pro microplate reader (TECAN) and in a Varioskan LUX multimode microplate reader (Thermo Scientific), respectively. Absorbance values were converted into mass of hydrolyzed nitrocefin using the Beer–Lambert Law and plotted over time. For each sample, the linear regression with the highest coefficient of determination was chosen and the quantity of nitrocefin hydrolyzed per min per mg of protein calculated. Assays were performed for each isolate with two biological replicates and the mean and the standard deviation were determined.

### Etbr accumulation and efflux assays

EtBr accumulation and efflux assays were performed as previously described (Rodrigues et al., 2015; Machado et al., 2017). Minimum inhibitory concentrations of EtBr and verapamil were obtained for *Msm* from broth microdilution assays as described above.

#### Accumulation assays

Cultures of *Msm* WT and selected resistant isolates were centrifuged at 3,000 *g* for 10 min. Supernatants were discarded and the pellets were resuspended in PBS supplemented with 0.05% Tween 80 and 0.4% glucose, and bacterial suspensions were normalized to an OD_600_ of 0.8. Aliquots of 100 μl of the suspensions were added to twofold serial dilutions of EtBr, previously prepared in black opaque 96-well plates, to obtain an EtBr dilution scale (0.125–8 mg/L). Fluorescence was measured every 60 s intervals for a period of 60 min in an Infinite M200 Pro microplate reader (TECAN), with parameters set at 530 nm excitation and 590 nm emission wavelengths. The steady-state concentration was determined as the concentration that resulted in a plateau of no more than 10% of relative fluorescence units achieved during the assay (Machado et al., 2017). Relative final fluorescence was calculated as the ratio of the fluorescence exhibited by each mutant to the fluorescence of the WT at the last timepoint of the respective steady-state concentration curves. Mean and standard deviation were calculated from two biological replicates.

#### Efflux assays

EtBr at steady-state concentration and verapamil at 1/4 of the MIC for each isolate, were added to *Msm* suspensions (OD_600_ = 0.8) prepared as stated in the previous section. After incubation for 1 h at 25°C with shaking, loaded cells were pelleted and resuspended in PBS supplemented with 0.05% of Tween 80. 100 μL of the suspensions were mixed with 100 μL of resuspension buffer with and without glucose and/or verapamil, added to final concentrations of 0.4% and 1/4 of the MIC, respectively. Cells were kept on ice to minimize EtBr efflux and fluorescence was measured in black opaque 96-well plates with an Infinite M200 Pro microplate reader (TECAN) (530 nm excitation; 590 nm emission), every 60 s for a period of 30 min. The obtained results are presented as relative fluorescence, obtained for each timepoint as the ratio of the fluorescence in the presence of glucose only or glucose plus verapamil, against data from the minimal efflux controls in the presence of verapamil with no glucose.

### WGS and association studies

Genomic DNA was extracted (Somerville et al., 2005) and quantified in a Qubit™ Fluorometer (Invitrogen). DNA samples were subjected to WGS using the Nextera XT Illumina library preparation protocol (Illumina), prior to paired-end sequencing (2x150bp or 2x250bp) on an Illumina MiSeq or NextSeq 550 equipment (Illumina), according to the manufacturer’s instructions.

The INNUca v4.2.2 pipeline^1^ was used for quality control and improvement of reads, species confirmation (using the 8GB database available at https://ccb.jhu.edu/software/kraken/) and bacterial *de novo* assembly. Draft genome sequences were annotated using the RAST server v2.0.^2^

Comparative whole-genome analysis of all isolates against *Mtb* H37Rv (GenBank accession number AL123456.3) or *Msm* mc^2^-155 (GenBank accession number CP000480.1) reference genomes, plus the analysis of all mutants versus the study WT and/or parental strains, allowed the identification of mutations possibly accountable for the observed phenotypes. The absence/presence of accessory genome was inspected through alignment of assemblies using the progressive algorithm of MAUVE v2.3.1 software.^3^ The identification of genetic markers was performed using Snippy v4.5.1 software.^4^ Essentially, quality improved reads of each *Mtb* or *Msm* mutant were individually mapped against the draft genome of the respective WT isolate. Variants were called on sites that filled the following criteria: i) minimum mapping quality and minimum base quality of 20; ii) minimum number of reads covering the variant position ≥10; and iii) minimum proportion of reads differing from the reference of 90%. All identified Single-nucleotide polymorphisms (SNPs) and insertion and deletion variants (indels) were carefully inspected and confirmed using IGV v2.12.3.^5^ Variants matching identified polymorphisms in different *Mtb* H37Rv strains (Ioerger et al., 2010) were not considered.

The affected genes, respective locus tag, and *Mtb* H37Rv orthologues, when described, were identified using the NCBI^6^ and Mycobrowser databases.^7^ SNPs and indels in *Mtb* target genes and their association with amoxicillin/clavulanate or meropenem/clavulanate susceptibility were obtained from available whole-genome sequences of clinical strains and their correspondent beta-lactam MICs (Olivença et al., 2022). To guarantee an appropriate number of strains with the wild type or mutant alleles, an inclusion criterion was set to consider only genomic variations detected in five or more strains diverging from all others. Mutations in clinical strains that obeyed the inclusion criterion and were previously associated with resistance to isoniazid or ethambutol were used as a control of the analysis. These included variants situated upstream *inhA* (*inhA*_c-777 t), in *katG* (*katG*_Ser315Thr), or in *embB* (*embB*_Met306Val; *embB*_Met306Ile) [World Health Organization (WHO), 2021].

### Data presentation and statistical analysis

Heatmaps and statistical analyses, including ANOVA and Kruskal–Wallis tests, were performed with GraphPad Prism version 9.0.

## Results

### Isolation of *Msm*-resistant mutants

To assess development of resistance in mycobacteria, the MICs of the antibiotics by the agar dilution method were firstly determined for mc^2^-155 and PM965, two strains of the non-pathogenic organism *Msm* (Table 1). The lowest MIC presented by *Msm* WT was to meropenem/clavulanate, while the highest were obtained to the anti-TB drugs rifampicin and isoniazid. For *Msm* PM965, no clavulanate was added to meropenem since this strain lacks BlaS (Raymond et al., 2005). However, the meropenem MIC of this mutant was fourfold higher than the observed for the WT. Resistant isolates were obtained by exposure to high antibiotic concentrations, equivalent to 5X, 10X and 20X the determined MIC. This process yielded isolates of mc^2^-155 resistant to amoxicillin, meropenem, vancomycin, isoniazid, rifampicin, and ethambutol. Resistant isolates of *Msm* PM965 were only obtained for meropenem. Isolation with anti-TB drugs mainly served as a validation of the method and, in the case of isoniazid and ethambutol, to produce double-resistant mutants from formerly obtained isolates as the parental strains. Except for PM965, all isolations with amoxicillin and meropenem were performed in the presence of clavulanate at 2.5 mg/L. In total, 33 *Msm* isolates were generated, which were named by the abbreviation(s) of the antibiotic(s) used to select them and the superscript letter “*R*”, followed by a roman number from I to III to identify the three isolates selected for each condition. In the case of the *Msm* PM965-derivatives, the name of the mutants is preceded by Δ*blaS*. A diagram of the several *Msm* mutants generated in this study and their parental strains is provided in Supplementary Figure S2A.

**Table 1:** Selection conditions and resistance frequency at which *Msm* isolates were retrieved.

Parental strain	Antibiotic	Agar dilution MIC (mg/L)	MIC multiple	Selection concentration (mg/L)	Plated CFUs	Output CFUs	Resistance frequency
WT	AMX/CLA	4	5x	20	1.00E+08	33	1.94E-07
			10x	40	1.00E+08	10	5.88E-08
			20x	80	1.00E+08	3	1.09E-08*
	MEM/CLA	0.5	10x	5	1.00E+07	16	2.72E-06
			20x	10	1.00E+08	8	1.36E-07*
					1.00E+09	50	8.52E-08
	VAN	8	10x	80	1.00E+08	4	2.35E-08
			20x	160	1.00E+08	14	8.24E-08*
	RIF	32	5x	160	1.00E+09	9	1.60E-08*
	INH	16	5x	80	1.00E+07	24	4.28E-06
					1.00E+08	66	1.18E-06
			10x	160	1.00E+07	6	1.07E-06
					1.00E+08	99	1.76E-06
			20x	320	1.00E+08	24	4.28E-07*
					1.00E+09	51	9.09E-08
	EMB	1	5x	5	1.00E+08	3	5.64E-08*
PM965	MEM	2	10x	20	1.00E+07	111	6.07E-06
			20x	40	1.00E+08	16	8.74E-08*
					1.00E+09	90	4.92E-08
INH*^R^* I	AMX/CLA*^a^*	4	10x	40	1.00E+07	7	1.27E-06
					1.00E+08	33	6.00E-07
			20x	80	1.00E+08	35	6.36E-07*
	MEM/CLA*^a^*	0.5	5x	2.5	1.00E+07	2	3.64E-07
					1.00E+08	2	3.64E-08
					1.00E+09	15	2.73E-08
			10x	5	1.00E+09	14	2.55E-08*
EMB*^R^* I	AMX/CLA*^b^*	4	5x	20	1.00E+07	88	2.13E-06
			10x	40	1.00E+07	77	1.86E-06
			20x	80	1.00E+07	84	2.03E-06*
	MEM/CLA*^b^*	0.5	5x	2.5	1.00E+08	3	7.25E-09
			10x	5	1.00E+09	16	3.86E-09
			20x	10	1.00E+09	8	1.93E-09*

Resistance frequency was determined in all conditions where colonies were visible and countable (*n* ≤ 150), and calculated as the ratio of number of colonies growing on antibiotic-containing plates to the total number of colony forming units (CFUs). Conditions from which the isolates were selected are marked with an asterisk (*). *^a^*The MICs of INH by the broth microdilution assay for the INH*^R^* AMX/CLA*^R^* and INH*^R^* MEM/CLA*^R^* mutants varied between 256–512 mg/L and 64–512 mg/L, respectively. *^b^*The MICs of EMB by the broth microdilution assay for the EMB*^R^* AMX/CLA*^R^* and EMB*^R^* MEM/CLA*^R^* isolates were 1 mg/L and 8 mg/L, respectively. AMX, amoxicillin; CLA, clavulanate; EMB, ethambutol; INH, isoniazid; MEM, meropenem; RIF, rifampicin VAN, vancomycin.

#### Resistance frequency

Resistance frequency was determined for all selection conditions where colonies were visible and countable (*n* ≤ 150) (Table 1). Resistance frequency ranged from 1.09E-8 to 1.94E-7 and from 8.52E-8 to 2.72E-6 for mutants selected from the WT after amoxicillin and meropenem pressure, respectively. PM965-derivatives selected with meropenem had slightly lower frequencies than those obtained for the WT, which may result from the higher meropenem concentrations employed on the selection from PM965. Frequency for vancomycin-selected isolates was below 1.00E-7.

For isoniazid, resistance frequency was between 9.09E-8 to 4.28E-6. These values are higher compared to the observed for amoxicillin-selected isolates, but similar to those obtained for meropenem-selected mutants. For rifampicin- and ethambutol-selected isolates, it was only possible to calculate the resistance frequency in one condition for each drug. Resistance frequency for both antibiotics was inferior to the calculated for isoniazid-resistant isolates in comparable selection conditions.

Regarding double-resistant isolates, resistance frequency for amoxicillin selection ranged from 6.00E-7 to 1.27E-6 for isolates stemming from INH^*R*^ I and from 1.86E-6 to 2.13E-6 for the ones derived from EMB^*R*^ I. In the case of meropenem-selection, frequencies varied between 2.55E-8 to 3.64E-7 and between 1.93E-9 and 7.25E-9 for isolates generated in the INH^*R*^ I and EMB^*R*^ I backgrounds, respectively.

#### MIC assays

**Figure 1:**
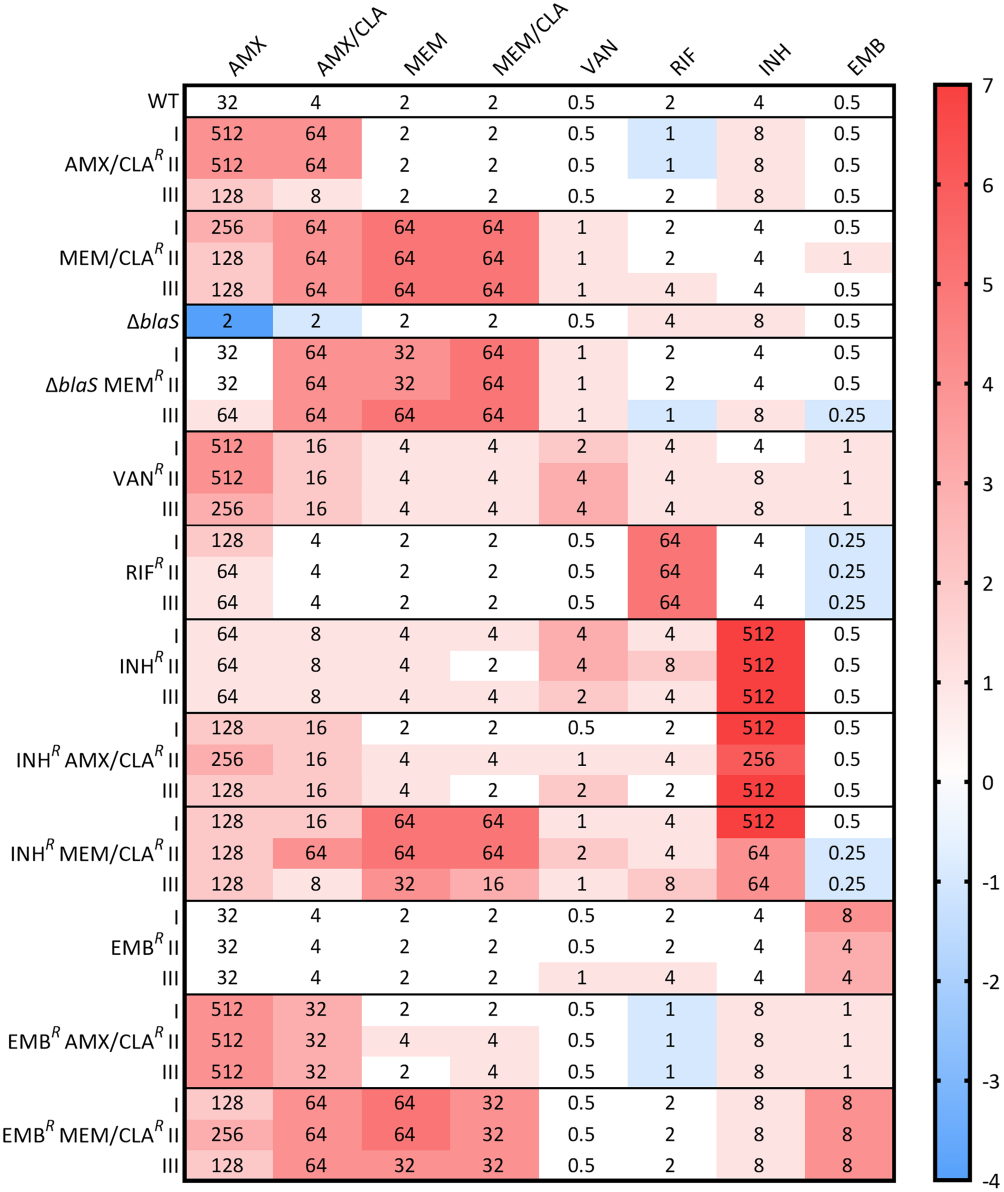
Heatmap of the log_2_ fold changes in liquid medium minimum inhibitory concentrations (MICs) of the resistant *Msm* mc^2^-155 isolates. Columns represent the antibiotics and rows represent the isolates. The median MIC in mg/L of each antibiotic for each isolate is shown in the respective intersection and colors depict an increase (red/pink shades) or decrease (blue shades) in the value when compared to the wild type (WT) MICs. Δ*blaS* corresponds to the PM965 strain and its derivatives. AMX, amoxicillin; CLA, clavulanate; EMB, ethambutol; INH, isoniazid; MEM, meropenem; RIF, rifampicin; VAN, vancomycin.

To confirm the resistance phenotype and to assess antibiotic susceptibility, the MICs of three PG synthesis inhibitors (amoxicillin and meropenem, with and without clavulanate, and vancomycin) and of three anti-TB drugs (rifampicin, isoniazid, and ethambutol) were determined for the resistant isolates and parental strains. The isolates MICs and log_2_ fold changes compared to the WT are presented in Figure 1. AMX/CLA*^R^* III had a lower increase in both amoxicillin and amoxicillin/clavulanate MICs when compared to the other two AMX/CLA*^R^* isolates. MEM/CLA*^R^* isolates exhibited an impressive log_2_ fold change of 5 in both meropenem and meropenem/clavulanate MICs. Significantly, these isolates were also more resistant to amoxicillin, especially when conjugated with clavulanate, revealing the development of cross-resistance between carbapenems and penicillins only after selection with meropenem. This prompted us to verify resistance to other beta-lactams, and therefore, MICs of three carbapenems (biapenem, doripenem, and ertapenem), a penem (faropenem), and a cephalosporin (cefotaxime), were additionally determined for AMX/CLA*^R^* I and MEM/CLA*^R^* I (Supplementary Table S1). MEM/CLA*^R^* I had an increase in the MICs to all beta-lactams, while AMX/CLA*^R^* I registered an increase in cefotaxime MICs, with or without clavulanate, and no considerable difference in the MIC of any carbapenem or faropenem.

As expected, PM965 was much more susceptible to amoxicillin. Like other meropenem-selected isolates, PM965-derivatives had log_2_ fold increases in beta-lactam MICs, with the amoxicillin MIC increase achieving the WT MIC values. In VAN*^R^* isolates, an increase in amoxicillin and amoxicillin/clavulanate MICs was detected in parallel with resistance to the glycopeptide itself.

Concerning selection with anti-TB drugs, isolates essentially presented resistance to the antibiotics used in the selection, but INH*^R^* mutants also had a MIC increase for vancomycin and a slight rise in almost all beta-lactam MICs. A major change in amoxicillin MICs was not observed for INH*^R^* AMX/CLA*^R^* isolates when compared to their parental strain. INH*^R^* MEM/CLA*^R^* mutants showed analogous results to other isolates selected with this carbapenem. Furthermore, all isolates generated from INH*^R^* I had a decrease in vancomycin MIC. Beta-lactam MICs of derivatives of EMB*^R^* I selected with amoxicillin or meropenem are mostly equivalent to the other isolates obtained in similar conditions. Interestingly, a decrease was observed in the ethambutol MIC of EMB*^R^* AMX/CLA*^R^* isolates, reverting to values similar to those exhibited by the WT.

### Isolation of *Mtb*-resistant mutants

We next sought to replicate the resistant mutant selection process in *Mtb* H37Rv (Supplementary Figure S2B). MICs by the agar dilution method for amoxicillin and meropenem, both with 2.5 mg/L of clavulanate, were determined as 2 and 1 mg/L, respectively. Agar plates with the beta-lactams added to a concentration 10X, 20X and 50X the MIC were prepared and concentrated *Mtb* suspensions were platted on these. Colonies were picked from the plates containing 50X the MIC determined by the agar dilution method and the lowest bacterial inoculum, as the other conditions usually yielded too many colonies to individually select them (Table 2). Additionally, while clavulanate was fixed at 2.5 mg/L when combined with meropenem, for amoxicillin selection it was increased to 25 mg/L to counterpose the more efficient hydrolysis of the penicillin by the potent BlaC of *Mtb*. Unlike *Msm*, in which all selected colonies were generally highly resistant to the drug used for isolation, a preliminary beta-lactam susceptibility screening showed that some *Mtb* isolates did not present phenotypic differences compared to the WT. These were discarded, while the isolates that showed at least a log_2_ fold increase of 2 in the MIC of the selection antibiotic in the preliminary assay were used in further assays and for genomic DNA extraction. Therefore, resistance frequency at which beta-lactam-resistant *Mtb* mutants were isolated could not be determined since it would be artificially increased by false positives. The increased challenges observed for isolation of *Mtb* mutants resistant to the beta-lactams may stem from several aspects: i) the required longer periods of incubation, during which the beta-lactam may degrade and allow growth of temporarily inhibited bacteria; ii) possible diffusion of beta-lactamase enzymes through the plate; iii) the lower division rate of *Mtb*, when compared with *Msm*, which may restrict the action of beta-lactams, usually more intense in actively replicating bacteria.

**Table 2:** Selection conditions used to obtain beta-lactam-resistant *Mtb* isolates.

Parental strain	Antibiotic	Agar dilution MIC (mg/L)	MIC multiple	Selection concentration (mg/L)	Plated CFUs
WT	AMX/CLA	2	10x	20	1.00E+07
					1.00E+08
					1.00E+09
			20x	40	1.00E+07
					1.00E+08
					1.00E+09
			50x	100	1.00E+07*
					1.00E+08
					1.00E+09
	MEM/CLA	1	10x	10	1.00E+07
					1.00E+08
					1.00E+09
			20x	20	1.00E+07
					1.00E+08
					1.00E+09
			50x	50	1.00E+07*
					1.00E+08
					1.00E+09

Conditions from which the isolates were selected are marked with an asterisk (*). AMX, amoxicillin; CLA, clavulanate; MEM, meropenem.

#### MIC assays

**Figure 2:**
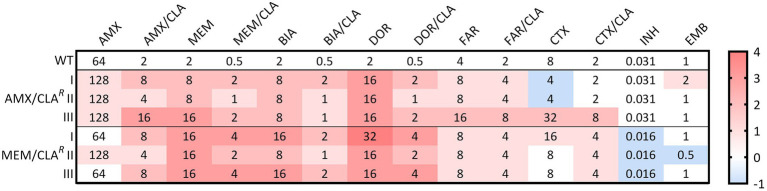
Heatmap of the log_2_ fold changes in liquid medium minimum inhibitory concentrations (MICs) of the resistant *Mtb* H37Rv isolates. Columns represent the antibiotics and rows represent the isolates. The median MIC in mg/L of each antibiotic for each isolate is shown in the respective intersection and colors depict an increase (red/pink shades) or decrease (blue shades) in the value when compared to the wild type (WT) MICs. AMX, amoxicillin; BIA, biapenem, CLA, clavulanate; CTX, cefotaxime; DOR, doripenem; EMB, ethambutol; FAR, faropenem; INH, isoniazid; MEM, meropenem.

The MICs of AMX/CLA*^R^* and MEM/CLA*^R^ Mtb* mutants were determined for isoniazid, ethambutol, and seven beta-lactams, with or without clavulanate (Figure 2). The most notorious MIC increases in *Mtb* were attained for carbapenems, particularly when not combined with clavulanate in MEM/CLA*^R^* isolates. Doripenem MICs had the highest log_2_ fold increases of 3 to 4, while the remaining carbapenems, with or without clavulanate, and amoxicillin/clavulanate had log_2_ fold changes ranging from 1 to 3. For unconjugated amoxicillin and both faropenem treatments, most isolates only attained a doubling of the MIC value. Cefotaxime results displayed less homogeneity, varying from a slight reduction to MICs four times higher than the WT. Isoniazid and ethambutol MICs were essentially unchanged, but all MEM/CLA*^R^* mutants had an isoniazid MIC that corresponded to half the value obtained for the parental strain.

#### Growth characterization

**Figure 3:**
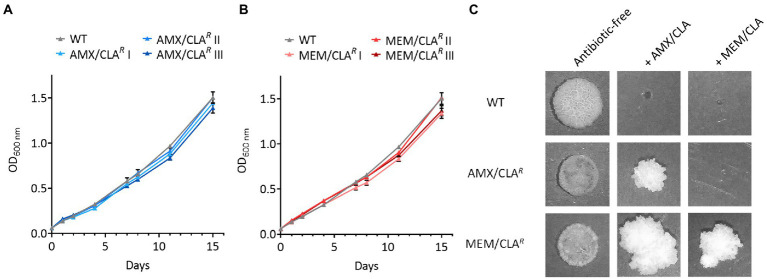
Growth of *Mtb*-resistant isolates and their parental strain in broth and agar medium. **(A)** Growth curves of the wild type (WT) and mutants selected with amoxicillin/clavulanate. **(B)** Growth curves of the WT and mutants selected with meropenem/clavulanate. Optical density at 600 nm (OD_600_) was measured over 15 days for all cultures of both groups of isolates. Triangles show the mean value of two biological replicates and error bars represent standard deviation. **(C)** Spots of the parental strain and one representative mutant of each group platted on antibiotic-free (11 days of incubation) and antibiotic-containing (>30 days of incubation) agar plates. The experiment was repeated twice giving comparable results. AMX, amoxicillin; CLA, clavulanic acid; MEM, meropenem.

Optical density measurements were performed over 15 days to detect possible growth differences between the resistant isolates and their parental strains. The growth curves of both AMX/CLA*^R^* and MEM/CLA*^R^* isolates overlapped the WT profile, namely up to timepoint 8 days (Figures 3A–B). After this, the mutants exhibited a small delay, more noticeable for MEM/CLA*^R^* I and III, but overall, the selection process with beta-lactams did not greatly impact growth in liquid medium.

To assess differences in solid medium, the WT and one isolate of the AMX/CLA*^R^* and MEM/CLA*^R^* groups were spotted onto antibiotic-free and antibiotic-containing plates, the latter with identical antibiotic concentrations to the ones used in the selection process. In accordance with the optical density curves, the isolates growth in antibiotic-free plates after 11 days was similar to their respective parental strain, albeit the spots had smaller dimensions and were less dense (Figure 3C). The mutants were able to survive in plates containing the antibiotic used for their isolation and no trace of the WT was detected. MEM/CLA*^R^* was additionally able to grow in the presence of high concentrations of amoxicillin/clavulanate, but the opposite was not observed. This is at odds with the disseminated beta-lactam cross-resistance shown in the *Mtb* MIC results but resonates with the findings of the same assay in *Msm*. One possible explanation for these phenotypic discrepancies may stem from a different relative importance of the mutated targets for growth in the two types of media, when supplemented with antibiotics. Similarly to the isolation procedure, growth in plates with beta-lactams required an extensive timeframe (>30 days) and yielded spots with a vastly different morphology, being more irregular and less flat, and also smaller in the case of AMX/CLA*^R^*.

### Contribution of beta-lactamase activity and cell wall permeability or efflux adaptations over the resistance phenotype

Since antibiotic inactivation by beta-lactamase hydrolysis is one of the resistance mechanisms used by mycobacteria against beta-lactams (Flores et al., 2005), this activity was assessed in *Msm* and *Mtb* resistant isolates through a nitrocefin hydrolysis assay. This quantification was performed for both cell lysate and supernatant samples from the WT and mutant strains. In *Msm*, PM965 and its meropenem-resistant derivatives were also studied. Antibiotic extrusion *via* efflux pumps is another common resistance mechanism used by bacteria against many classes of antimicrobial agents, including beta-lactams (Rodrigues et al., 2020). To assess the influence of this mechanism in the resistance phenotype, the accumulation and efflux of EtBr across the cell wall was determined for the *Msm* resistant isolates selected with a beta-lactam antibiotic and the parental WT strain, in the presence and absence of verapamil, an efflux inhibitor.

#### Beta-lactamase assays

**Figure 4:**
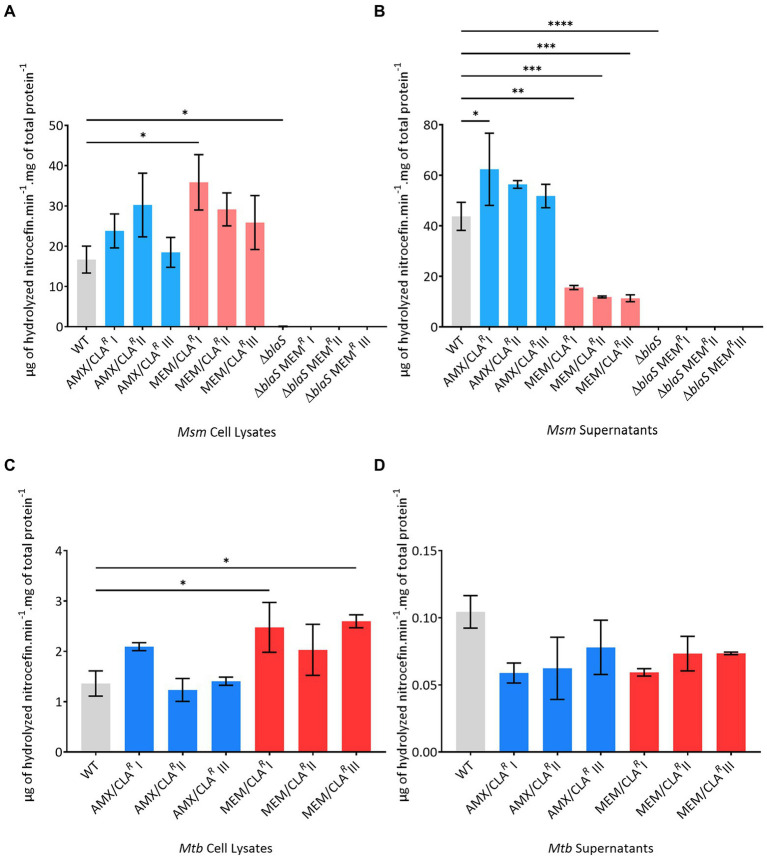
Beta-lactamase activity of the *Msm* and *Mtb* parental strains or resistant isolates, selected with amoxicillin or meropenem. **(A)** *Msm* cell lysates. **(B)** *Msm* supernatants. **(C)** *Mtb* cell lysates. **(D)** *Mtb* supernatants. Beta-lactamase activity is expressed as μg of hydrolyzed nitrocefin per min per mg of total protein. The assay was performed with two biological replicates for each isolate. Mean values were calculated and error bars express standard deviations. The significance of the differences between samples of resistant derivatives and the wild type (WT) was determined using an ANOVA test: *when *p* ≤ 0.05; ** when *p* ≤ 0.01; ***when *p* ≤ 0.001; ****when *p* ≤ 0.0001. AMX, amoxicillin; CLA, clavulanic acid; MEM, meropenem.

Nitrocefin hydrolysis in the cell lysate samples of *Msm* AMX/CLA*^R^* and MEM/CLA*^R^* mutants was generally higher than the obtained for the WT (Figure 4A), but statistically significant differences were only noted for the isolate MEM/CLA*^R^* I (*p* value = 0.012). A similar pattern of increased activity was observed for the supernatants of the amoxicillin-selected isolates (Figure 4B). On the contrary, MEM/CLA*^R^* isolates consistently showed a very low beta-lactamase activity in the supernatants (*p* values ≤0.0012). As anticipated, *Msm* PM965 and its meropenem-selected isolates exhibited null activity levels in both supernatants and cell lysates.

The beta-lactamase activity in cell lysate samples of *Mtb* H37Rv (Figure 4C) was much lower than the measured for *Msm* WT. All MEM/CLA*^R^ Mtb* isolates and AMX/CLA*^R^* I presented increased cell-associated activities, with MEM/CLA*^R^* I and III surpassing the double of the parental strain mean value (*p* value ≤0.047). Distinctly from *Msm*, the hydrolytic activity in the supernatant of *Mtb* WT was lower than in the cell lysate (Figure 4D). Hydrolysis in the supernatants of *Mtb* beta-lactam-resistant mutants was even more reduced, and despite being transversal to all isolates, this reduction was not significant as in the *Msm* MEM/CLA*^R^* mutants.

#### Accumulation and efflux assays

**Figure 5:**
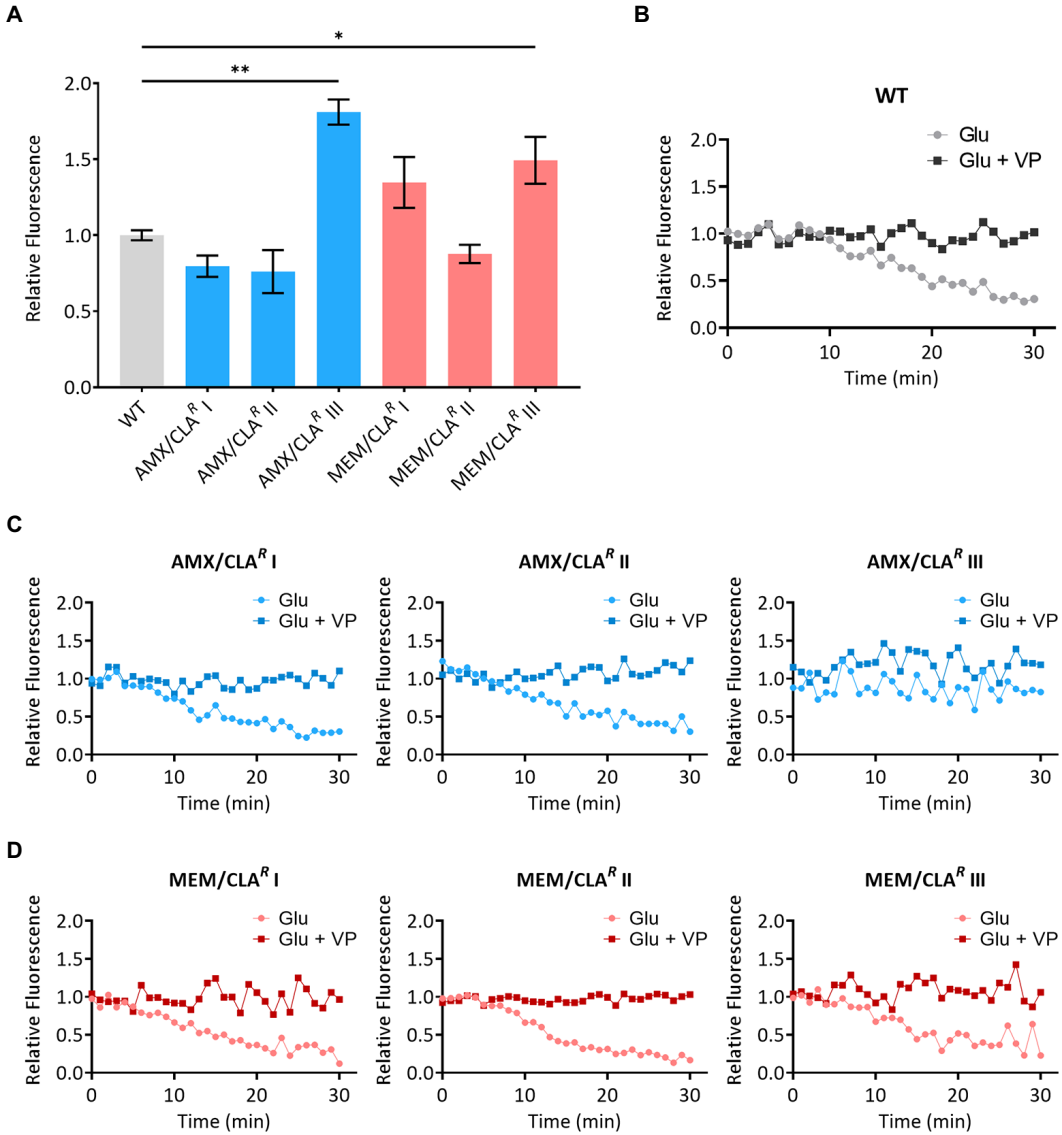
Accumulation and efflux of ethidium bromide by *Msm* parental strain and beta-lactam-resistant mutants. **(A)** Mean relative fluorescence at 60 min was calculated from assays with two biological replicates for each strain and error bars express standard deviations. The significance of the differences between samples of resistant derivatives and the wild type (WT) was determined using an ANOVA test: *when *p* ≤ 0.05; **when *p* ≤ 0.01. **(B)** Efflux activity of the WT and resistant mutants isolated with amoxicillin/clavulanate **(C)** or meropenem/clavulanate **(D)**, in the presence of glucose (Glu) or glucose plus verapamil (VP). AMX, amoxicillin; CLA, clavulanic acid; MEM, meropenem.

The MICs of EtBr and the efflux inhibitor verapamil were firstly determined to ensure cellular viability would not be compromised in the accumulation and efflux assays (Supplementary Table S2). No major differences were observed between *Msm* isolates and the respective parental strain, with EtBr and verapamil MICs ranging from 1 to 2 mg/L and from 256 to 512 mg/L, respectively. We then performed the accumulation assays to establish the EtBr steady-state concentration at which uptake and efflux rates are at equilibrium in each strain (Supplementary Table S2). The steady-state concentration of the WT and three isolates (AMX/CLA*^R^* I, AMX/CLA*^R^* II, and MEM/CLA*^R^* II) was 0.25 mg/L. The remaining mutants had a steady-state concentration of 0.125 mg/L. The mean relative final fluorescence and respective error values at the 60 min timepoint of the steady-state concentration curves are presented in Figure 5A. The isolates with the lower steady-state concentration (AMX/CLA*^R^* III, MEM/CLA*^R^* I, and MEM/CLA*^R^* III) had a higher accumulation of EtBr. These observations suggest these mutants present some alteration in either the efflux activity or permeability of the cell wall.

Afterward, bacteria were loaded with EtBr to access efflux activity. Overall, a decrease in the relative fluorescence in the presence of glucose was observed for most strains, corresponding to the EtBr efflux (Figures 5B–D). When verapamil was added to glucose, relative fluorescence remained constant overtime, confirming that the inhibitor was able to effectively block the efflux in all strains. Among the isolates with higher EtBr accumulation than the WT, AMX/CLA*^R^* III had a comparable relative fluorescence in both conditions, showing a restricted efflux activity even in the absence of verapamil. While this may be responsible for the altered EtBr accumulation phenotype of this mutant, since there was no significant efflux reduction in MEM/CLA*^R^* I and III, their enhanced accumulation may otherwise be related with changes in cell wall permeability.

### Mutations associated with resistance phenotypes to peptidoglycan synthesis inhibitors

Besides changes in cell wall permeability and beta-lactamase or efflux pump activities, other strategies bacteria use to overcome the effects of antibiotics include the modification of drug targets or the alteration of their expression. Therefore, a genomic analysis of selected *Msm* and *Mtb* isolates was performed to identify chromosomal mutations possibly involved in these resistance mechanisms to beta-lactams and vancomycin.

#### WGS of *Msm* beta-lactam- and vancomycin-resistant isolates

Sequencing of the 33 *Msm* antibiotic-resistant isolates yielded 24 different mutations, spanning 19 genomic locus or intragenic regions (Supplementary Table S3). A compilation of the mutations found in all *Msm* beta-lactam- or vancomycin-resistant mutants and their characteristics is shown in Table 3. These consisted of 20 distinct mutations, which occurred in 19 genomic locus or intragenic regions. AMX/CLA*^R^* and EMB*^R^* AMX/CLA*^R^* isolates shared mutations in genes encoding conserved hypothetical protein MSMEG_0317 and the IclR family transcriptional regulator MSMEG_3335. They also had an intergenic insertion between *MSMEG_5710* and *MSMEG_5711* in common with the VAN*^R^* mutants. Differently from the other isolates of the group, AMX/CLA*^R^* III had SNPs in *MSMEG_5732* and *MSMEG_5450*, which are translated into the sensor protein KdpD and the redox-sensitive transcriptional activator SoxR, respectively. All VAN*^R^* isolates had a mutation in *MSMEG_5487*, a sensor histidine kinase gene. One of these mutants additionally carried the same *MSMEG_0317* and *MSMEG_3335* mutations described above. Almost all mutants resistant to meropenem had an intergenic mutation 8 or 9 bp upstream the transcription start site (TSS) of *MSMEG_6319*. Importantly, this gene encodes a penicillin-binding protein/transpeptidase, orthologue of *Rv2864c* in *Mtb*. MEM/CLA*^R^* I and III were the only meropenem-selected mutants without this mutation, instead displaying an intergenic mutation 11 bp before the TSS of *MSMEG_6317*, the gene adjacent to *MSMEG_6319* and which produces a lipolytic enzyme. The fact that AMX/CLA*^R^* III and MEM/CLA*^R^* II exhibited unique mutations compared with the other two isolates of the respective groups, may account for the differences observed in the nitrocefin hydrolysis and EtBr accumulation/efflux assays.

**Table 3:** Mutations and affected genes identified on *Msm* isolates selected with beta-lactams or vancomycin.

Isolate group	# of isolates with mutation	Genome position*^a^*	Type*^b^*	Nt change*^c^*	AA change*^d^*	Locus tag or upstream region	Product	Mtb H37Rv orthologue*^e^*
AMX/CLA*^R^*	3/3	351,880	snp	A191C	Glu64Ala	MSMEG_0317	Conserved hypothetical protein	Rv0227c
	3/3	3,413,225	snp	T785A	Ile262Asn	MSMEG_3335	Transcript. Regulator, IclR family protein	(Rv2989)
	1/3	5,450,148	snp	C1582T	Arg528Cys	MSMEG_5372	Sensor protein KdpD	(Rv1028c)
	1/3	5,535,781	snp	G140A	Arg47His	MSMEG_5450	Redox-sensitive transcript. Activator SoxR	–
	3/3	5,795,546	ins	G > GCC	intergenic	–215 bp from TSS of MSMEG_5710	Hypothetical protein	–
MEM/CLA*^R^*	2/3	6,382,809	snp	G > A	intergenic	–11 bp from TSS of MSMEG_6317	Lipolytic enzyme, G-D-S-L	–
	1/3	6,383,472	snp	G > A	intergenic	–9 bp from TSS of MSMEG_6319	Penicillin-binding protein, transpeptidase	(Rv2864c)
Δ*blaS* MEM*^R^*	3/3	6,383,472	snp	G > A	intergenic	–9 bp from TSS of MSMEG_6319	Penicillin-binding protein, transpeptidase	(Rv2864c)
VAN*^R^*	1/3	351,880	snp	A191C	Glu64Ala	MSMEG_0317	Conserved hypothetical protein	Rv0227c
	1/3	3,413,225	snp	T785A	Ile262Asn	MSMEG_3335	Transcript. Regulator, IclR family protein	(Rv2989)
	3/3	5,569,289	complex	241_247delCTCATCGinsTGCAGCT	LeuIleGlu81*	MSMEG_5487	Sensor histidine kinase MprB	Rv0982
	3/3	5,795,546	ins	G > GCC	intergenic	–215 bp from TSS of MSMEG_5710	Hypothetical protein	–
INH*^R^* AMX/CLA*^R^*	2/3	522,537	ins	11dupC	Arg5fs	MSMEG_0448	Transcript. Regulator, MarR family protein	–
	1/3	647,961	snp	C > T	intergenic	–119 bp from TSS of MSMEG_0574	Putative ECF sigma factor RpoE1	–
	3/3	1,014,410	ins	645dupC	Gly216fs	MSMEG_0933	D-inositol-3-phosphate glycosyltransferase MshA	Rv0486
	1/3	1,039,095	snp	T188C	Leu63Pro	MSMEG_0965	Porin	–
	3/3	4,464,789	ins	G > GT	intergenic	–40 bp from TSS of MSMEG_4378	Two-component system response regulator	–
INH*^R^* MEM/CLA*^R^*	1/3	522,221	snp	C186T	Ala62Ala	MSMEG_0447	Conserved hypothetical protein	–
	3/3	522,806	snp	C274T	Arg92Cys	MSMEG_0448	Transcript. Regulator, MarR family protein	–
	3/3	1,014,410	ins	645dupC	Gly216fs	MSMEG_0933	D-inositol-3-phosphate glycosyltransferase MshA	Rv0486
	3/3	4,464,789	ins	G > GT	intergenic	–40 bp from TSS of MSMEG_4378	Two-component system response regulator	–
	2/3	6,383,472	snp	G > A	intergenic	–9 bp from TSS of MSMEG_6319	Penicillin-binding protein, transpeptidase	(Rv2864c)
	1/3	6,383,473	snp	C > T	intergenic	–8 bp from TSS of MSMEG_6319	Penicillin-binding protein, transpeptidase	(Rv2864c)
	3/3	6,853,426	snp	A1391C	Asp464Ala	MSMEG_6805	Beta-lactamase	(Rv1730c)
EMB*^R^* AMX/CLA*^R^*	3/3	351,880	snp	A191C	Glu64Ala	MSMEG_0317	Conserved hypothetical protein	Rv0227c
	3/3	3,413,225	snp	T785A	Ile262Asn	MSMEG_3335	Transcript. Regulator, IclR family protein	(Rv2989)
	3/3	5,795,546	ins	G > GCC	intergenic	–215 bp from TSS of MSMEG_5710	Hypothetical protein	–
EMB*^R^* MEM/CLA*^R^*	3/3	3,157,252	snp	C > G	intergenic	–25 bp from TSS of MSMEG_3084	Glyceraldehyde-3-phosphate dehydrogenase Gap	Rv1436
	3/3	5,795,546	ins	G > GCC	intergenic	–215 bp from TSS of MSMEG_5710	Hypothetical protein	–
	3/3	6,383,472	snp	G > A	intergenic	–9 bp from TSS of MSMEG_6319	Penicillin-binding protein, transpeptidase	(Rv2864c)
	3/3	6,451,445	snp	G920A	Arg307His	MSMEG_6389	Probable arabinosyltransferase A	Rv3795
	1/3	6,861,902	snp	T > C	intergenic	–21 bp from TSS of MSMEG_6811	Conserved hypothetical protein	–

a Genome position corresponds to the sequence coordinate on the *Msm* mc^2^-155 reference genome (GenBank accession number CP000480.1). *^b^*ins, insertion; snp, single nucleotide polymorphism. *^c^*In the cases where the mutation occurred in a coding region, the nucleotide (Nt) position within the gene is shown. *^d^*fs, translation frameshift; *, translation termination. *^e^*Orthologue product according to Mycobrowser; orthologues predicted with BLASTp tool are shown between parentheses; −, not available. AMX, amoxicillin; CLA, clavulanate; EMB, ethambutol; INH, isoniazid; MEM, meropenem; transcript., transcriptional; TSS, transcription start site; VAN, vancomycin.

**Figure 6:**
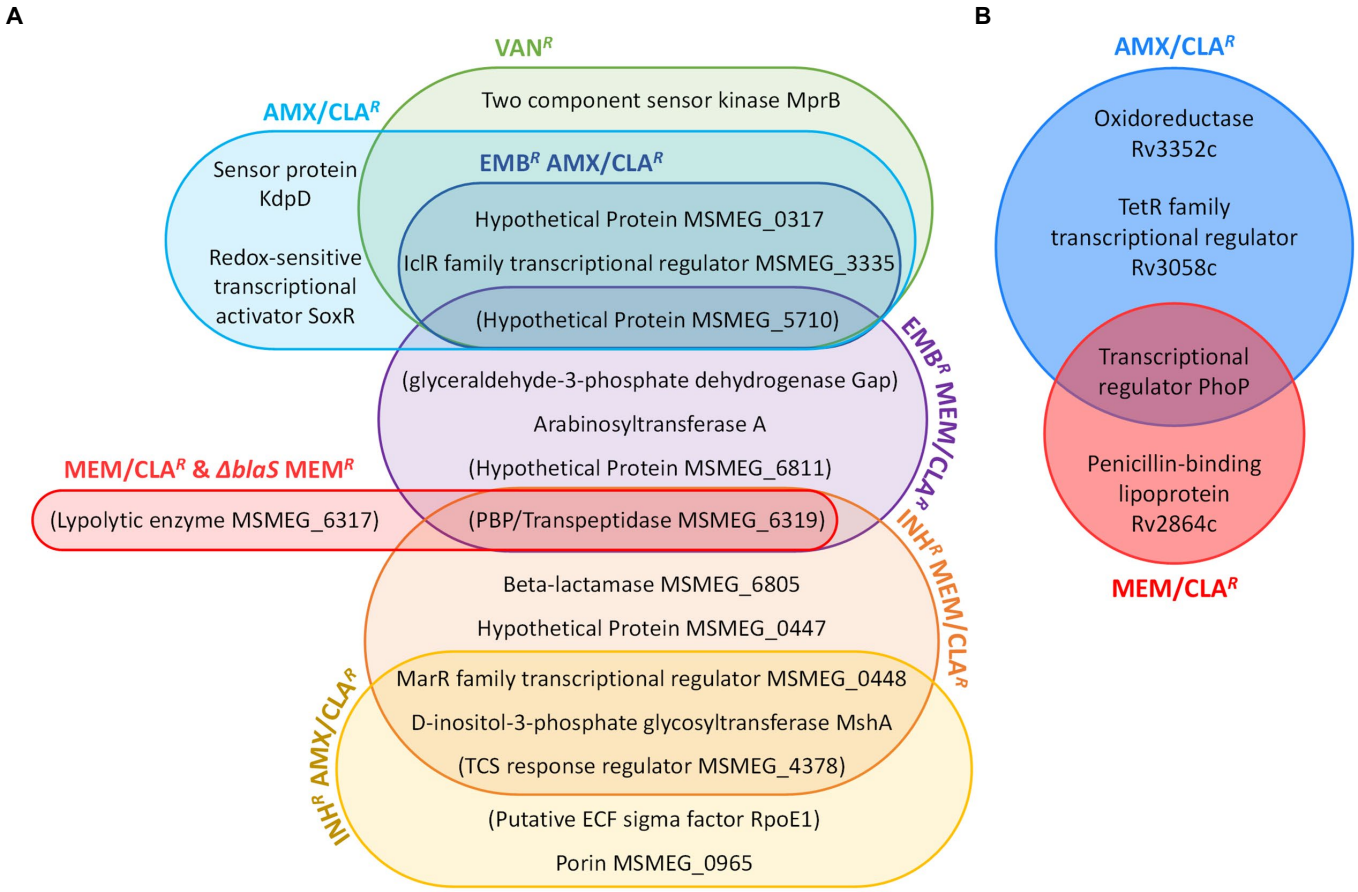
Interplay of determinants of resistance to peptidoglycan synthesis inhibitors. **(A)** Diagram of beta-lactam- and vancomycin-resistance in *Msm*. **(B)** Diagram of beta-lactam-resistance in *Mtb*. Determinants are represented in both diagrams by the name of the product in which genomic mutations were, respectively, identified. In the case of intergenic mutations, the name of the product encoded by the gene with the closest transcription start site is represented inside brackets. AMX, amoxicillin; CLA, clavulanate; EMB, ethambutol; INH, isoniazid; MEM, meropenem; TCS, two-component system; VAN, vancomycin.

Sequencing of PM965 (Δ*blaS* in Supplementary Table S3) revealed that, in addition to the disruption of the beta-lactamase gene, this strain had more seven mutations when compared with our laboratory WT. These may be partially responsible for the differences in agar dilution meropenem MICs displayed by the two strains. Despite this, meropenem-resistant mutants isolated from PM965 did not carry any additional mutations besides the one located upstream the TSS of *MSMEG_6319*. On the contrary, double-resistant isolates generally had a more complex mutational profile than the beta-lactam-selected single mutants. Five out of the six beta-lactam-resistant mutants obtained from an isoniazid-resistant parental strain had a mutation in the gene of a MarR family transcriptional regulator (*MSMEG_0448*). INH*^R^* MEM/CLA*^R^* isolates additionally possessed a mutation in *MSMEG_6805*, a beta-lactamase gene orthologue of *Rv1730c* and annotated as encoding a possible PBP in *Mtb*, while EMB*^R^* MEM/CLA*^R^* mutants had an intergenic mutation upstream the *gap* gene, encoding for a glyceraldehyde-3-phosphate dehydrogenase. Other mutations were only detected in one isolate of the double-resistant mutants. The most relevant of these includes a SNP occurring within the porin-encoding gene *MSMEG_0965* in an INH*^R^* AMX/CLA*^R^* isolate. A graphic summary of the interplay of key determinants found in beta-lactam- and vancomycin-resistant *Msm* mutants is provided in Figure 6A.

According to the expected, mutations in the isolates selected with an anti-TB drug occurred in genes targeted by these antibiotics and already described in *Mtb*, validating our selection methodology. The rifampicin-selected isolates all presented a mutation in the *rpoB* gene (*MSMEG_1367*), which encodes the β-subunit of the DNA-dependent RNA polymerase. Albeit distinct, all isoniazid-selected isolates had mutations that resulted in either premature translation termination or translation frameshift of MshA, a D-inositol-3-phosphate glycosyltransferase involved in mycothiol biosynthesis. The *mshA* gene is not targeted by isoniazid, but mutations in genes involved in mycothiol synthesis were associated with increased resistance to isoniazid and ethionamide in both *Msm* and *Mtb* (Xu et al., 2011; Vilchèze and Jacobs, 2014). Ethambutol-selected isolates presented mutations in *MSMEG_6389*, an arabinosyltransferase orthologue to *embB* of *Mtb*. EMB*^R^* AMX/CLA*^R^* isolates lost the Arg307His substitution of the parental strain, leading to the decreased ethambutol resistance observed for these mutants after amoxicillin exposure.

#### WGS of *Mtb* beta-lactam-resistant isolates

*Mtb* mutants had a more homogeneous pattern of genomic variants within each group (Figure 6B). AMX/CLA*^R^* isolates had mutations in *Rv3352c* and *Rv3058c*, which encode an oxidoreductase and a TetR-family transcriptional regulator, respectively (Table 4; Supplementary Table S4). All beta-lactam-resistant mutants presented a *phoP* (*Rv0757*) mutation. Additionally, *Mtb* MEM/CLA*^R^* mutants showed a mutation in *Rv2864c*, a gene encoding for a penicillin-binding lipoprotein. In the pathogen, this variant results in a synonymous mutation, rather than a modification in a probable promoter region like described for *Msm* meropenem-selected mutants.

**Table 4:** Mutations and affected genes identified on *Mtb* isolates selected with beta-lactams.

Isolate group	# of isolates with mutation	Genome position^a^	Type*^b^*	Nt change*^c^*	AA change	Locus tag	Product
AMX/CLA*^R^*	2/3	851,902	snp	A295G	Thr99Ala	Rv0757	Possible two component system response transcriptional positive regulator PhoP
	1/3	852,106	snp	A499G	Lys167Glu	Rv0757	Possible two component system response transcriptional positive regulator PhoP
	3/3	3,418,847	snp	C530T	Thr177Ile	Rv3058c	Possible transcriptional regulatory protein (probably TetR-family)
	3/3	3,768,564	snp	C30G	Val10Val	Rv3352c	Possible oxidoreductase
MEM/CLA*^R^*	3/3	852,161	snp	T554C	Phe185Ser	Rv0757	Possible two component system response transcriptional positive regulator PhoP
	3/3	3,175,952	snp	C1314T	Ala438Ala	Rv2864c	Possible penicillin-binding lipoprotein

a Genome position corresponds to the sequence coordinate on the *Mtb* H37Rv reference genome (GenBank accession number AL123456.3). *^b^*snp, single nucleotide polymorphism. *^c^*In the cases where the mutation occurred in a coding region, the nucleotide (Nt) position within the gene is shown. AMX, amoxicillin; CLA, clavulanate; MEM, meropenem.

### Correlation of mutations in putative beta-lactam resistance target genes with amoxicillin/clavulanate and meropenem/clavulanate MICs in *Mtb* clinical strains

An association between mutations in *Mtb* genes identified by WGS of the beta-lactam-resistant mutants and the phenotypic data obtained for a set of clinical *Mtb* strains was performed. *Mtb* and *Msm* main mutations related with meropenem-resistance were associated with orthologue genes (Tables 3; 4). The remaining identified drivers of beta-lactam resistance were mostly transcriptional regulators, sensor kinases or proteins involved in redox reactions or balance in both model organisms (Figure 6). Given this correspondence, we decided to include mutations in *Mtb* genes that are orthologues to those identified in *Msm*, allowing an extended pool of interest targets. This analysis aimed to verify if strains carrying variations in these genes would have significantly different susceptibilities to amoxicillin and meropenem, both with the beta-lactamase inhibitor clavulanate, when compared to strains with the WT allele.

Out of the four genes revealed by WGS in *Mtb*, clinical strains had mutations in *Rv2864c* and *Rv3058c*. Of these, only two mutations in *Rv2864c* complied with the defined inclusion criterion. At least one mutation of the clinical strains that conformed to this condition was also found for all the *Mtb* orthologues (*Rv0227c*, *Rv1028c*, *Rv1436*, *Rv1730c*, *Rv2864c*, and *Rv2989*) of *Msm* genes or gene promoter regions with mutations identified in the WGS of the resistant isolates.

**Figure 7:**
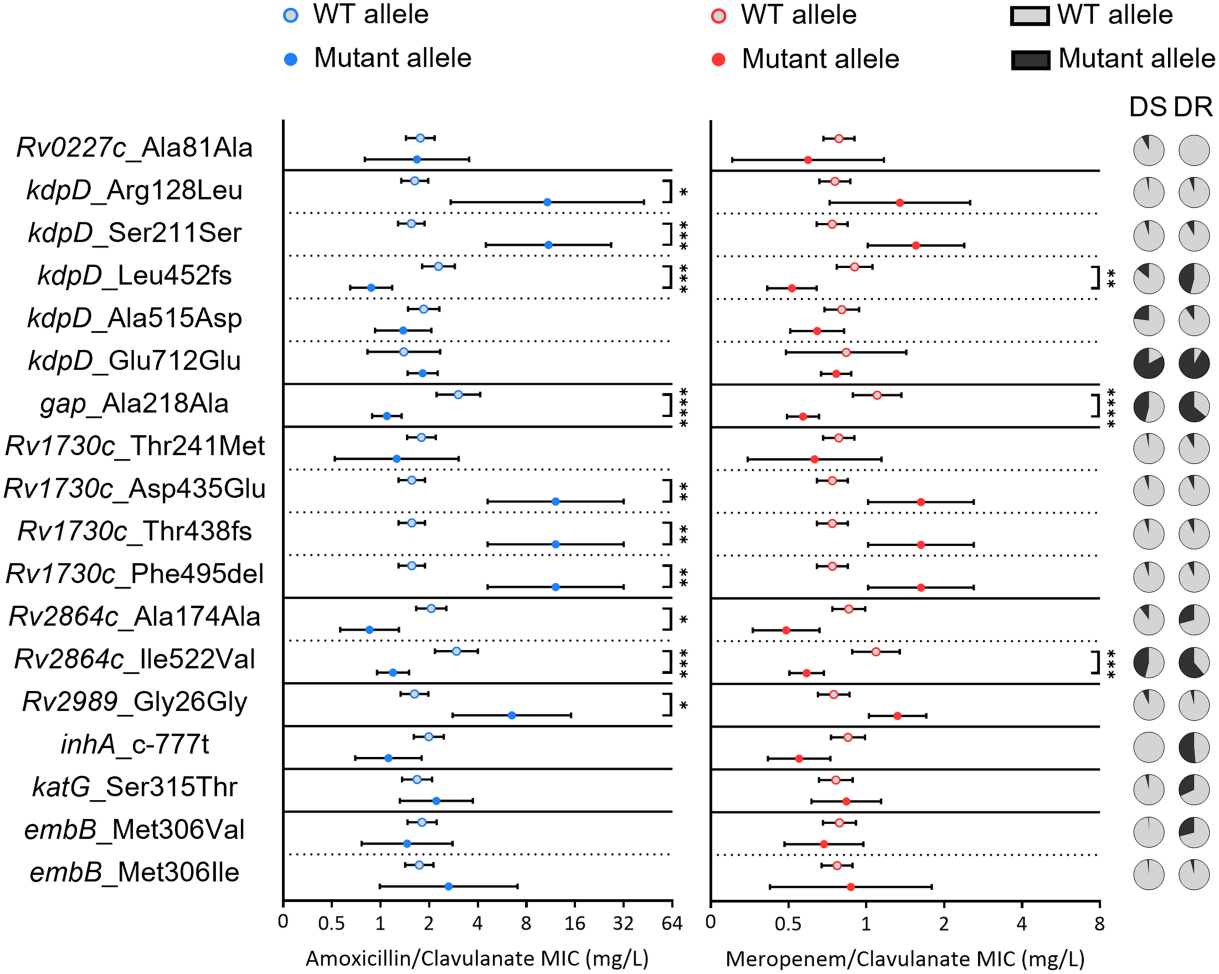
Beta-lactam susceptibility of *Mtb* clinical strains with mutations in relevant target or orthologue genes. The geometric mean minimum inhibitory concentration of amoxicillin/clavulanate (blue) or meropenem/clavulanate (red) for clinical strains carrying the wild type (WT) (gray dots with color outline) or mutant allele (full color dots) is depicted for each considered amino acid substitution. Error bars show a 95% confidence interval. A Kruskal–Wallis test was performed to evaluate statistically significant differences between the two groups for each mutation: *when *p* ≤ 0.05; **when *p* ≤ 0.01; ***when *p* ≤ 0.001; ****when *p* ≤ 0.0001. Pie charts represent the distribution of each mutation in anti-TB drug-susceptible (DS) and drug-resistant (DR) strains.

Clinical strains with mutations in KdpD (Arg128Leu and Ser211Ser), Rv2989 (Gly26Gly) and Rv1730c (all except for Th241Met) had an amoxicillin/clavulanate mean MIC significantly higher than strains without these substitutions (Figure 7). On the other hand, the frameshift in Leu452 of KdpD, the mutation in Gap and both Rv2864c mutations were linked to lower MICs to this penicillin. Excluding the synonymous substitution in Rv2864c, the presence of the remaining mutations in clinical strains was also associated with statistically significant increased meropenem/clavulanate susceptibility. Notably, these three mutations were more frequent in clinical strains with previous resistance to anti-TB drugs, while most other mutations in genes under study had equal representation in both susceptible- and drug-resistant isolates. Regarding the control mutations in *embB*, *katG* or in the promoter of the *fabG1*-*inhA* operon [World Health Organization (WHO), 2021], no meaningful difference in beta-lactam MICs was detected between strains with and without these variants.

## Discussion

In this work, we challenged mycobacteria with high concentrations of beta-lactams or vancomycin and then performed genomic and phenotypic studies on the obtained mutants, to better understand how these bacteria develop resistance to PG synthesis inhibitors and to anticipate possible genomic markers of beta-lactam resistance or susceptibility in clinical *Mtb* strains.

While the increase in beta-lactam MICs was less pronounced for *Mtb* mutants, cross-resistance to antibiotics of this class was also noted after selection with amoxicillin and not only after incubation with meropenem, as detected in the *Msm* isolates. Both amoxicillin and meropenem target PBPs, while Ldts are not considered to be inhibited by amoxicillin (Story-Roller and Lamichhane, 2018). We hypothesized that the observed phenotypes could result from mutations in genes encoding Ldts or major PBPs, like PonA1, PonA2 or PbpA, which could lead to reduced or loss of affinity for the beta-lactams. However, the WGS analyses revealed that neither *Mtb* nor *Msm* had mutations in these genes. Instead, *Mtb* mutants had a mutation within the sequence of *Rv2864c* and *Msm* isolates presented a mutation closely upstream the corresponding orthologue gene *MSMEG_6319*. These variants were associated with high-level resistance to meropenem in both organisms. Rv2864c is a putative high-molecular-mass PBP with a lipoprotein motif, recently shown to be relatively resistant to all beta-lactam subclasses when compared to other well-known PBPs (Kumar et al., 2022). The mutation in the putative promoter region of *MSMEG_6319* (AT701_RS30910) has been previously described in meropenem-resistant *Msm* mutants (Maeda et al., 2021). The detection of a mutation in *Rv2864c* in the meropenem-resistant *Mtb* isolates in our study is in accordance with these observations and further supported by reports of increased cefotaxime sensitivity in a *Mtb* mutant generated through *Rv2864c* disruption and by CRISPR interference studies on *Mycobacterium abscessus* orthologue gene *MAB_3167c* (Wivagg et al., 2016; Akusobi et al., 2022). Maeda et al. (2021) additionally report in their meropenem-selected mutants a non-synonymous mutation in the coding sequence of *MSMEG_6319* and an intergenic variant upstream *MSMEG_6406* (AT701_RS31385), orthologue of Rv3811 and which encodes a putative N-acetylmuramoyl-l-alanine amidase. We did not observe the two latter features, but two of our meropenem-exposed mutants alternatively displayed a mutation 11 bp upstream the TSS of *MSMEG_6317*, adjacent to *MSMEG_6319*.

Noteworthy, we have identified mutations in PhoP in all of our *Mtb* beta-lactam-selected mutants. PhoPR is a two-component regulatory system that regulates essential genes for complex lipid biosynthesis, affecting *Mtb* CW composition and virulence (Walters et al., 2006). Like other response regulators of these systems, PhoP has a N-terminal regulatory domain and a C-terminal effector domain (Menon and Wang, 2011). In our work, we show that mutations in AMX/CLA*^R^* II and III mutants (Thr99Ala) lie within the regulatory domain, while amino acid substitutions in AMX/CLA*^R^* I (Lys167Glu) and in MEM/CLA*^R^* isolates (Phe185Ser) occur in the C-terminal domain. BlrAB, a two-component regulatory system of *Aeromonas* Spp., has been shown to play a part in the regulation of the expression of the beta-lactamase (Niumsup et al., 2003). Hence, the increased cell-associated beta-lactamase activity observed for these four mutants may be linked to mutations arising within the domain that interacts with the cellular transcription machinery. Variants in Rv3058c, another putative transcriptional regulator, and in the oxidoreductase Rv3352c were also found in amoxicillin-resistant *Mtb* mutants. In parallel, several transcriptional regulators and proteins involved in redox sensing or function were identified in the AMX/CLA*^R^ Msm* mutants. These findings are highly pertinent since they resonate with evidence on the role played by the cytoplasmic redox potential and the transcription factor WhiB4 for amoxicillin/clavulanate resistance in *Mtb* (Mishra et al., 2017).

Although vancomycin does not directly inhibit the transpeptidases, a *Mtb* strain lacking both *ldt_Mt1_* and *ldt_Mt2_* has shown enhanced susceptibility to amoxicillin/clavulanate and vancomycin (Schoonmaker et al., 2014). Since the target of this glycopeptide is the dipeptide D-Ala-D-Ala, resistance to this drug could trigger alterations that influence beta-lactam susceptibility. Accordingly, our *Msm* VAN*^R^* mutants displayed cross-resistance to amoxicillin and most of the mutations found in these isolates coexisted in the amoxicillin-resistant isolates. This suggests that resistance to these antibiotics in this species may be more related than between the two beta-lactams used in our study. Nonetheless, all VAN*^R^* mutants possessed a specific mutation in *MrpB*, which resulted in a premature translation termination. MprB is a membrane associated protein involved in the MprAB system, another two-component regulatory system described in *Mtb* (Zahrt et al., 2003), further supporting the importance of these systems for resistance to PG synthesis inhibitors. Evidence of cross-resistance between beta-lactams and anti-TB was not detected, but INH*^R^* mutants had vancomycin MIC fold increases comparable with the vancomycin-selected *Msm* isolates. Since isoniazid and vancomycin are, respectively, small and large hydrophilic molecules, this observation may stem from adjustments in transport of water-soluble compounds or CW permeability.

The fact that the *Mtb* MEM/CLA*^R^* mutants showed similar levels of resistance to the ones displayed by isolates selected with amoxicillin suggests that the main driver of beta-lactam resistance phenotypes may actually be PhoP. However, the additional synonymous mutation in Rv2864c seemed to be a key determinant for survival of the MEM/CLA*^R^* mutant in agar containing meropenem, which could be linked to an altered expression rate of this protein. Therefore, the role of all identified players and their potential interactions within the mechanism of action of beta-lactams in *Mtb* should be clearly asserted and its clinical significance investigated. We show that clinical strains harboring the Ile522Val substitution within the transpeptidase motif (PF00905, E-value 2.1E-35) of Rv2864c were significantly more susceptible to both amoxicillin and meropenem with clavulanate. Importantly, this mutation was more prevalent in drug-resistant strains, which fits earlier reports of paradoxical beta-lactam hypersusceptibility among certain MDR and extensively drug-resistant *Mtb* isolates (Cohen et al., 2016; Olivença et al., 2022). *Msm* double-resistant mutants were isolated at a higher frequency to amoxicillin/clavulanate but at a lower frequency for meropenem/clavulanate than the corresponding isolates obtained from the WT. In general, the double mutants carried additional mutations and had a more diverse mutational profile, but that did not reflect in superior beta-lactam MIC compared with the WT-derivatives. This implies that the capacity to develop resistance to beta-lactams may be influenced by preexistent resistance to CW inhibitors isoniazid and ethambutol, which should be further explored in *Mtb*.

Beta-lactamase inducible systems are common in bacteria and there is evidence suggesting that this mechanism can be found in *Mtb* (Sala et al., 2009). In our study, the cell lysate beta-lactamase activity was considerably higher in the WT *Msm* than in *Mtb*. This observation and the respective nitrocefin hydrolysis values are in line with previously reported results (Flores et al., 2005). As described by these authors, the majority of the beta-lactamase of the pathogen was cell-associated. This is in opposition with earlier studies that mention increased activity in culture broth, rather than in cell pellets (Voladri et al., 1998), like we observed for *Msm*. Overall, *Msm* mutants had an increase in the cell lysate activity, which was also noted in *Mtb* selected with meropenem. On the other hand, supernatant activity was reduced in *Msm* MEM/CLA*^R^*, and to a much lesser extent, in all *Mtb*-resistant isolates. Although increased expression might occur, these discrepancies are suggestive of a decrease in beta-lactamase extrusion, possibly in an attempt to retain it within the cell. BlaS secretion into the periplasm of *Msm* is dependent of a twin-arginine translocase system, with mutants deficient for proteins involved in this pathway showing alterations in secretion of beta-lactamase and an increase in cell-associated activity (McDonough et al., 2005; Posey et al., 2006). Mutations that restrict the flow of the beta-lactamase to the exterior of the cell, by affecting CW permeability or unknown translocators, may result in the observed distribution shift. Since transpeptidation occurs in the periplasm, concentration of the beta-lactamase in this compartment could help the bacteria cope with increased concentrations of beta-lactams. In PM965, BlaS deficiency considerable impaired its resistance to amoxicillin. While *Msm* has an accessory beta-lactamase enzyme, BlaE, hypothesized to be a cephalosporinase (Flores et al., 2005), alongside other putative beta-lactamases (Bansal et al., 2017), their activity was not detected, even in the meropenem-selected isolates. Moreover, Δ*blaS* MEM/CLA*^R^* isolates had MIC increases comparable to the yielded by MEM/CLA*^R^* mutants. An exception was noted for amoxicillin, for which mutants appear to require the presence of BlaS to exceed the values of the WT MIC. This indicates that beta-lactam resistance exhibited by our isolates is not exclusively dependent on increased beta-lactamase activity and that exposure to different sub-groups of beta-lactams might lead to the involvement of different mechanisms of resistance.

In general, the *Msm* isolates showed few variations in EtBr accumulation or efflux compared to the parental strains, with some exceptions where alterations in the interaction between permeability and efflux were suggested. Apparent alterations in CW permeability were detected in *Msm* MEM/CLA*^R^* I and III, the same mutants that contain a mutation upstream *MSMEG_6317*, annotated as encoding a lipolytic enzyme. Since essentially no MIC differences between these isolates and other meropenem-selected mutants were detected, the mutated region could actually correspond to the promoter of a putative *MSMEG_6317*-*MSMEG_6319* operon and also affect the expression of the downstream gene. Increased accumulation of EtBr in these mutants may have resulted from altered activity of this enzyme over the lipid network of *Msm* to better accommodate the predicted lipoprotein MSMEG_6319. In addition, the porin content may influence the permeability of the mycobacterial CW and *Msm* mutants lacking the major porin MspA have been associated with higher MICs to both beta-lactams and vancomycin (Danilchanka et al., 2008). Mutations in porin genes were absent in *Mtb*. In *Msm*, a Glu64Ala substitution in MSMEG_0317 was found for AMX/CLA*^R^*, EMB*^R^* AMX/CLA*^R^* and VAN*^R^* mutants. The product of this gene includes a predicted porA chanel-forming protein motif (PF11271, E-value 3.5E-96), which suggests MSMEG_0317 might be involved in transport of these antibiotics. Regarding efflux pumps, some of these systems reported in *Mtb* are resistance nodulation division transporters (Rodrigues et al., 2020). The PG layer has a role in stabilizing one of these transporters in *Pseudomonas aeruginosa* and *Escherichia coli* (Ma et al., 2021). Thus, alterations in the PG structure of the *Msm* AMX/CLA*^R^* III mutant due to amoxicillin exposure could have resulted in unstable efflux pump anchorage and, by consequence, its unexpectedly decreased efflux capacity. Since our study was based on exposure to high antibiotic concentrations, the contribution of these systems to beta-lactam resistance in other conditions, such as an adaptation to subinhibitory concentrations (Garima et al., 2015), should not be discarded.

In conclusion, our study outputs imply that mycobacterial resistance to beta-lactams may not be as simplistic as once thought. Confirmation of mutations in *Rv2864c* and upstream *MSMEG_6319* in mutants isolated with meropenem, corroborated previous descriptions of the involvement of the *Msm* gene in resistance to this carbapenem (Maeda et al., 2021). This is also in agreement with our previous description that *Mtb* clinical strains with a mutation in LpqK, another lipoprotein with similarity to PBPs and D,D-carboxypeptidases, show reduced meropenem MICs (Olivença et al., 2022). Thus, our findings in *Mtb* H37Rv and clinical strains are consistent with a more prominent role to what is currently attributed to lesser-known (lipo)proteins with putative PBP/beta-lactamase activity (Kumar et al., 2022). Importantly, although amoxicillin and meropenem are members of the same antibiotic class, both model organisms developed resistance by acquiring mutations in different genes, with exception of *phoP* in *Mtb*. Thus, at least two distinct pathways may potentially impact beta-lactam resistance in mycobacteria. Other transcription regulators, specially two-component systems, as well as redox activity (Mishra et al., 2017), emerge as possible key determinants of high beta-lactam resistance. RNA sequencing and analysis of the transcriptome of *Mtb* WT and its mutants would offer further insights into the interplay of the identified genes and further contribute to understand PG synthesis dynamics upon beta-lactam exposure. Combined with validation of their role, this additional information could lead to the development of novel compounds aimed at these potential targets, thereby blocking PG synthesis, or potentiating the effects of current inhibitors.

## Data availability statement

The datasets presented in this study can be found in online repositories. Raw sequencing reads of all strains and derivative drug-resistant mutants of *Mtb* H37Rv and *Msm* mc^2^-155 were deposited in the European Nucleotide Archive (ENA) under the BioProject accession number PRJEB53916. Individual accession numbers are available in Supplementary Table S5.

## Author contributions

FO and MJC conceived and designed the study. FO, CF, AN, and CS performed the experiments. FO, CF, AN, CS, MP, JPG, and MJC analyzed the data. FO wrote the manuscript. All authors contributed to the article and approved the submitted version.

## Funding

This work was supported by the European Society of Clinical Microbiology and Infectious Diseases (ESCMID), Switzerland, through Research Grant 2018, and by Fundação para a Ciência e a Tecnologia (FCT), Portugal, through research project PTDC/BIA-MIC/31233/2017, awarded to MJC. FO (SFRH/BD/136853/2018) and CS (2021.05446.BD) are recipients of PhD fellowships from FCT.

## Conflict of interest

The authors declare that the research was conducted in the absence of any commercial or financial relationships that could be construed as a potential conflict of interest.

## Publisher’s note

All claims expressed in this article are solely those of the authors and do not necessarily represent those of their affiliated organizations, or those of the publisher, the editors and the reviewers. Any product that may be evaluated in this article, or claim that may be made by its manufacturer, is not guaranteed or endorsed by the publisher.

## Footnotes

^1^ https://github.com/B-UMMI/INNUca

^2^ http://rast.nmpdr.org

^3^ http://darlinglab.org/mauve/mauve.html

^4^ https://github.com/tseemann/snippy

^5^ http://software.broadinstitute.org/software/igv/

^6^ https://www.ncbi.nlm.nih.gov/

^7^ https://mycobrowser.epfl.ch/
