# Supplementary material for: Identification of drivers of mycobacterial resistance to peptidoglycan synthesis inhibitors
Source: Front Microbiol. 2022 Sep 6;13:985871. doi: 10.3389/fmicb.2022.985871 (PMC9485614; doi:10.3389/fmicb.2022.985871)
Supplement: Supplementary file 1 [file Data_Sheet_1.PDF]

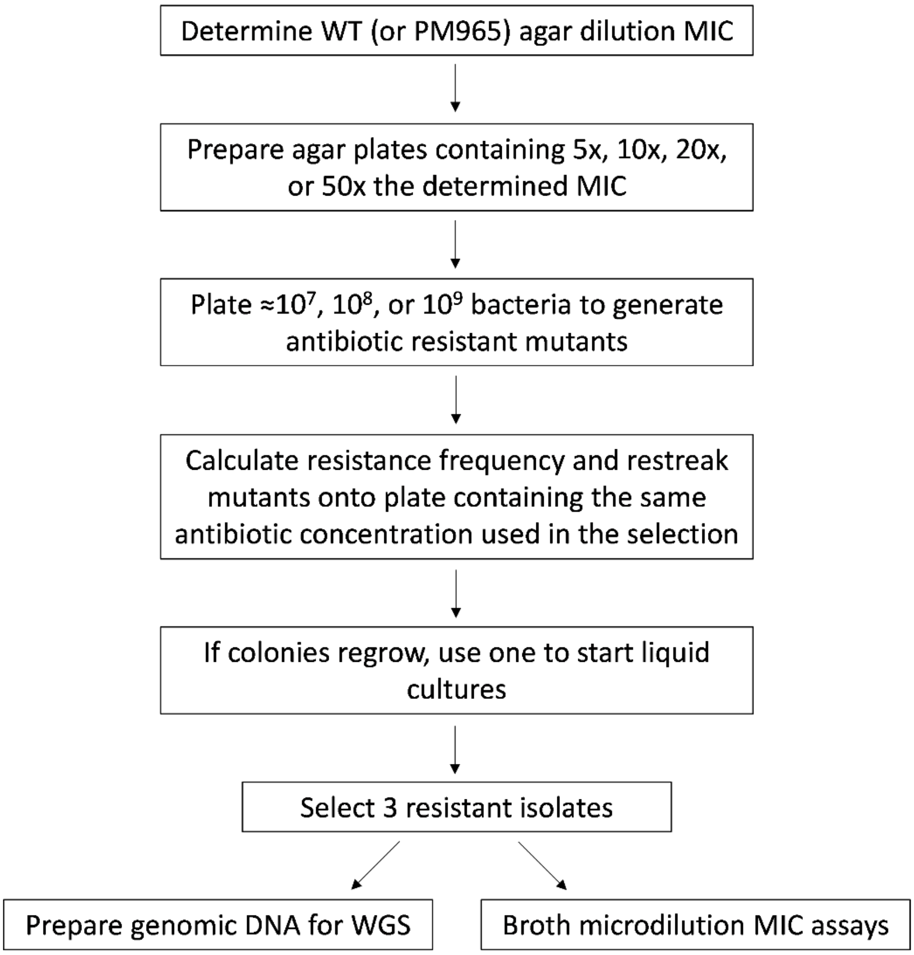

Figure S1. Schematic representation of the resistant mutant isolation process applied in this study.

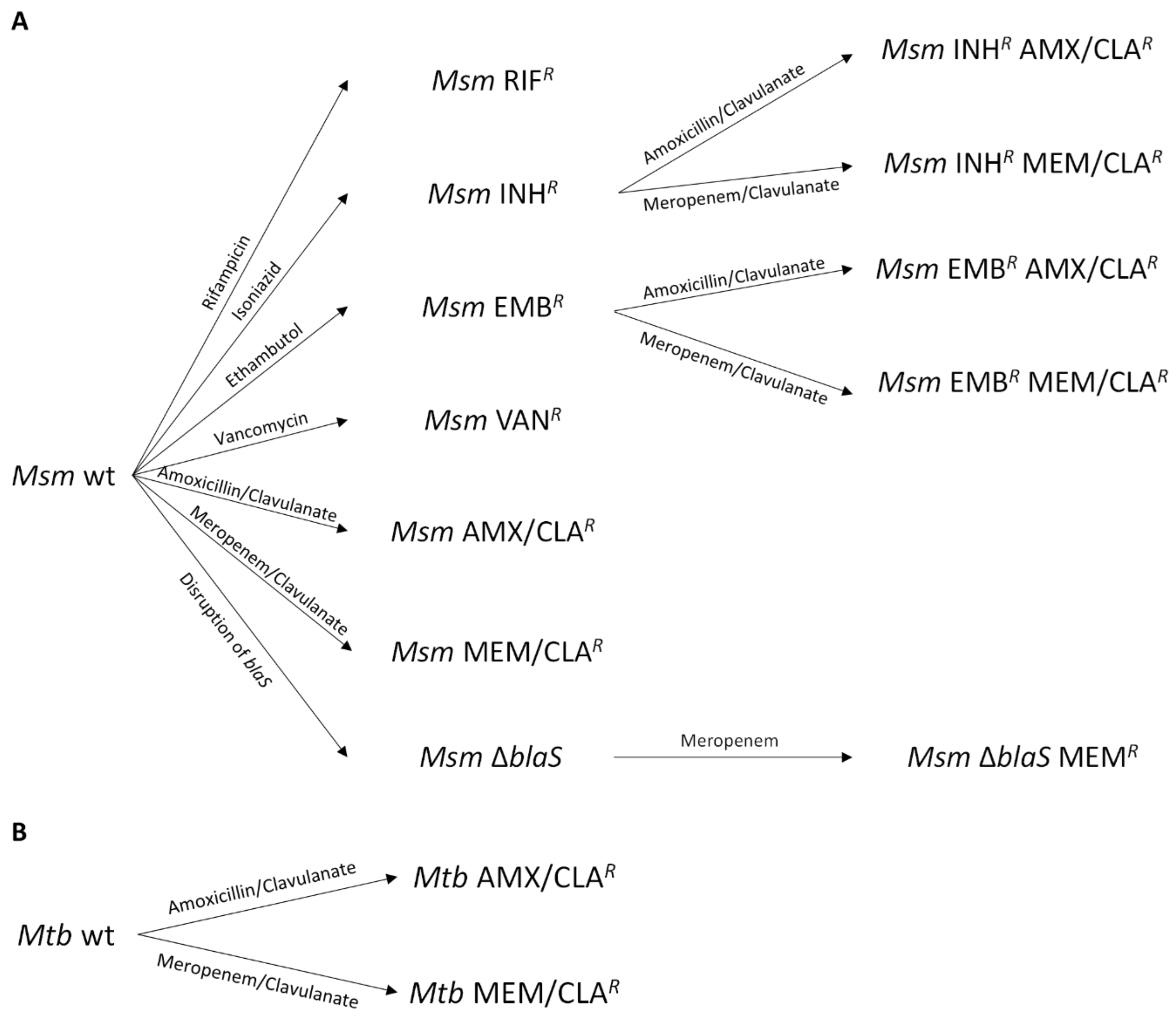

Figure S2. Schematic diagrams depicting the evolutionary tree for *Msm* and *Mtb* mutant strain isolation. (A) Mutants generated from *Msm*. (B) Mutants generated from *Mtb*.

Table S1. Minimum inhibitory concentrations (mg/L) of carbapenems, faropenem and cefotaxime, with and without 2.5 mg/L of clavulanate, obtained for *M. smegmatis* beta-lactam-resistant isolates and wild type (WT) parental strain. The obtained results are presented as the mean of three assays. AMX, amoxicillin; BIA, biapenem; CLA, clavulanate; CTX, cefotaxime; DOR, doripenem; ETP, ertapenem; FAR, faropenem; MEM, meropenem.

	BIA	DOR	ETP	FAR	CTX	CTX/CLA
WT	2	2	8	4	64	64
AMX/CLA^R I	4	2	16	8	512	512
MEM/CLA^R I	64	32	128	16	512	512

Table S2. Verapamil (VP) and ethidium bromide (EtBr) minimum inhibitory concentration (MIC) and steady-state concentration (SSC) obtained for *M. smegmatis* resistant isolates and respective wild type (WT) parental strain. The obtained results are presented as the mean of at least two assays. AMX, amoxicillin; CLA, clavulanate; MEM, meropenem.

	MIC		EtBr SSC
	VP	EtBr	
WT	512	2	0.25
AMX/CLA^R I	512	2	0.25
AMX/CLA^R II	512	2	0.25
AMX/CLA^R III	512	2	0.125
MEM/CLA^R I	256	2	0.125
MEM/CLA^R II	512	1	0.25
MEM/CLA^R III	512	2	0.125

Table S3. Mutations and affected genes identified on all *Msm* isolates of this study.

[a] Genome position corresponds to the sequence coordinate on the *Msm* mc^2-155 reference genome (GenBank accession number CP000480.1). [b] ins, insertion; snp, single nucleotide polymorphism. [c] In the cases where the mutation occurred in a coding region, the nucleotide (Nt) position within the gene is shown. [d] fs, translation frameshift; *, translation termination. [e] Mutations besides the disruption of the *blaS* gene are shown. [f] In addition to the mutation shown, this mutant also presents the mutations found for the strain Δ*blaS* . AMX, amoxicillin; CLA, clavulanate; EMB, ethambutol; INH, isoniazid; MEM, meropenem; RIF, rifampicin; TSS, transcription start site; VAN, vancomycin.

Isolate	Genome position[a]	Type[b]	Nt change[c]	AA change[d]	Locus tag or upstream region	Product
Δ*blaS*[e]	1498429	snp	A128G	Lys43Arg	MSMEG_1398	ribosomal protein S12
	2739477	snp	G1167A	Ala389Ala	MSMEG_2657	peptidase, M16 family protein
	3687351	ins	28dupC	Leu10fs	MSMEG_3624	urease accessory protein uref
	3973795	snp	G>A	intergenic	-528bp from TSS of MSMEG_3901	L-2,4-diaminobutyric acid acetyltransferase
	5420607	snp	C>T	intergenic	-523bp from TSS of MSMEG_5332	transcriptional regulator, TetR family protein
	6635553	snp	T507A	Asn169Lys	MSMEG_6583	antigen 85-C
	6691973	snp	T1007C	Leu336Pro	MSMEG_6641	nitrilotriacetate monooxygenase component A
AMX/CLA^R I	351880	snp	A191C	Glu64Ala	MSMEG_0317	conserved hypothetical protein
	3413225	snp	T785A	Ile262Asn	MSMEG_3335	transcriptional regulator, IclR family protein
	5795546	ins	G>GCC	intergenic	-215bp from TSS of MSMEG_5710	hypothetical protein
AMX/CLA^R II	351880	snp	A191C	Glu64Ala	MSMEG_0317	conserved hypothetical protein
	3413225	snp	T785A	Ile262Asn	MSMEG_3335	transcriptional regulator, IclR family protein
	5795546	ins	G>GCC	intergenic	-215bp from TSS of MSMEG_5710	hypothetical protein
AMX/CLA^R III	5450148	snp	C1582T	Arg528Cys	MSMEG_5372	sensor protein KdpD
	5535781	snp	G140A	Arg47His	MSMEG_5450	redox-sensitive transcriptional activator SoxR
MEM/CLA^R I	6382809	snp	G>A	intergenic	-11bp from TSS of MSMEG_6317	lipolytic enzyme, G-D-S-L
MEM/CLA^R II	6383472	snp	G>A	intergenic	-9bp from TSS of MSMEG_6319	penicillin-binding protein, transpeptidase
MEM/CLA^R III	6382809	snp	G>A	intergenic	-11bp from TSS of MSMEG_6317	lipolytic enzyme, G-D-S-L
Δ*blaS* MEM^R I[f]	6383472	snp	G>A	intergenic	-9bp from TSS of MSMEG_6319	penicillin-binding protein, transpeptidase
Δ*blaS* MEM^R II[f]	6383472	snp	G>A	intergenic	-9bp from TSS of MSMEG_6319	penicillin-binding protein, transpeptidase
Δ*blaS* MEM^R III[f]	6383472	snp	G>A	intergenic	-9bp from TSS of MSMEG_6319	penicillin-binding protein, transpeptidase
VAN^R I	351880	snp	A191C	Glu64Ala	MSMEG_0317	conserved hypothetical protein
	3413225	snp	T785A	Ile262Asn	MSMEG_3335	transcriptional regulator, IclR family protein
	5569289	complex	241_247delCTCATCGinsTGCAGCT	LeuIleGlu81*	MSMEG_5487	sensor histidine kinase MprB
	5795546	ins	G>GCC	intergenic	-215bp from TSS of MSMEG_5710	hypothetical protein
VAN^R II	5569289	complex	241_247delCTCATCGinsTGCAGCT	LeuIleGlu81*	MSMEG_5487	sensor histidine kinase MprB
	5795546	ins	G>GCC	intergenic	-215bp from TSS of MSMEG_5710	hypothetical protein
VAN^R III	5569289	complex	241_247delCTCATCGinsTGCAGCT	LeuIleGlu81*	MSMEG_5487	sensor histidine kinase MprB
	5795546	ins	G>GCC	intergenic	-215bp from TSS of MSMEG_5710	hypothetical protein
RIF^R I	1462954	snp	A1325G	His442Arg	MSMEG_1367	DNA-directed RNA polymerase, beta subunit
RIF^R II	1462954	snp	A1325G	His442Arg	MSMEG_1367	DNA-directed RNA polymerase, beta subunit
RIF^R III	1462954	snp	A1325G	His442Arg	MSMEG_1367	DNA-directed RNA polymerase, beta subunit
INH^R I	1014410	ins	645dupC	Gly216fs	MSMEG_0933	D-inositol-3-phosphate glycosyltransferase MshA
	4464789	ins	G>GT	intergenic	-40bp from TSS of MSMEG_4378	two-component system response regulator
INH^R II	1014979	snp	C1209G	Tyr403*	MSMEG_0933	D-inositol-3-phosphate glycosyltransferase MshA
INH^R III	1013795	ins	31dupC	Arg11fs	MSMEG_0933	D-inositol-3-phosphate glycosyltransferase MshA
INH^R AMX/CLA^R I	522537	ins	11dupC	Arg5fs	MSMEG_0448	transcriptional regulator, MarR family protein
	1014410	ins	645dupC	Gly216fs	MSMEG_0933	D-inositol-3-phosphate glycosyltransferase MshA
	4464789	ins	G>GT	intergenic	-40bp from TSS of MSMEG_4378	two-component system response regulator
INH^R AMX/CLA^R II	1014410	ins	645dupC	Gly216fs	MSMEG_0933	D-inositol-3-phosphate glycosyltransferase MshA
	1039095	snp	T188C	Leu63Pro	MSMEG_0965	porin
	4464789	ins	G>GT	intergenic	-40bp from TSS of MSMEG_4378	two-component system response regulator
INH^R AMX/CLA^R III	522537	ins	11dupC	Arg5fs	MSMEG_0448	transcriptional regulator, MarR family protein
	647961	snp	C>T	intergenic	-119bp from TSS of MSMEG_0574	putative ECF sigma factor RpoE1
	1014410	ins	645dupC	Gly216fs	MSMEG_0933	D-inositol-3-phosphate glycosyltransferase MshA
	4464789	ins	G>GT	intergenic	-40bp from TSS of MSMEG_4378	two-component system response regulator
INH^R MEM/CLA^R I	522221	snp	C186T	Ala62Ala	MSMEG_0447	conserved hypothetical protein
	522806	snp	C274T	Arg92Cys	MSMEG_0448	transcriptional regulator, MarR family protein
	1014410	ins	645dupC	Gly216fs	MSMEG_0933	D-inositol-3-phosphate glycosyltransferase MshA
	4464789	ins	G>GT	intergenic	-40bp from TSS of MSMEG_4378	two-component system response regulator
	6383472	snp	G>A	intergenic	-9bp from TSS of MSMEG_6319	penicillin-binding protein, transpeptidase
	6853426	snp	A1391C	Asp464Ala	MSMEG_6805	beta-lactamase
INH^R MEM/CLA^R II	522806	snp	C274T	Arg92Cys	MSMEG_0448	transcriptional regulator, MarR family protein
	1014410	ins	645dupC	Gly216fs	MSMEG_0933	D-inositol-3-phosphate glycosyltransferase MshA
	4464789	ins	G>GT	intergenic	-40bp from TSS of MSMEG_4378	two-component system response regulator
	6383472	snp	G>A	intergenic	-9bp from TSS of MSMEG_6319	penicillin-binding protein, transpeptidase
	6853426	snp	A1391C	Asp464Ala	MSMEG_6805	beta-lactamase
INH^R MEM/CLA^R III	522806	snp	C274T	Arg92Cys	MSMEG_0448	transcriptional regulator, MarR family protein
	1014410	ins	645dupC	Gly216fs	MSMEG_0933	D-inositol-3-phosphate glycosyltransferase MshA
	4464789	ins	G>GT	intergenic	-40bp from TSS of MSMEG_4378	two-component system response regulator
	6383473	snp	C>T	intergenic	-8bp from TSS of MSMEG_6319	penicillin-binding protein, transpeptidase
	6853426	snp	A1391C	Asp464Ala	MSMEG_6805	beta-lactamase
EMB^R I	6451445	snp	G920A	Arg307His	MSMEG_6389	probable arabinosyltransferase A
EMB^R II	6451441	snp	T916C	Phe306Leu	MSMEG_6389	probable arabinosyltransferase A
EMB^R III	6451445	snp	G920A	Arg307His	MSMEG_6389	probable arabinosyltransferase A
EMB^R AMX/CLA^R I	351880	snp	A191C	Glu64Ala	MSMEG_0317	conserved hypothetical protein
	3413225	snp	T785A	Ile262Asn	MSMEG_3335	transcriptional regulator, IclR family protein
	5795546	ins	G>GCC	intergenic	-215bp from TSS of MSMEG_5710	hypothetical protein
EMB^R AMX/CLA^R II	351880	snp	A191C	Glu64Ala	MSMEG_0317	conserved hypothetical protein
	3413225	snp	T785A	Ile262Asn	MSMEG_3335	transcriptional regulator, IclR family protein
	5795546	ins	G>GCC	intergenic	-215bp from TSS of MSMEG_5710	hypothetical protein
EMB^R AMX/CLA^R III	351880	snp	A191C	Glu64Ala	MSMEG_0317	conserved hypothetical protein
	3413225	snp	T785A	Ile262Asn	MSMEG_3335	transcriptional regulator, IclR family protein
	5795546	ins	G>GCC	intergenic	-215bp from TSS of MSMEG_5710	hypothetical protein
EMB^R MEM/CLA^R I	3157252	snp	C>G	intergenic	-25bp from TSS of MSMEG_3084	glyceraldehyde-3-phosphate dehydrogenase Gap
	5795546	ins	G>GCC	intergenic	-215bp from TSS of MSMEG_5710	hypothetical protein
	6383472	snp	G>A	intergenic	-9bp from TSS of MSMEG_6319	penicillin-binding protein, transpeptidase
	6451445	snp	G920A	Arg307His	MSMEG_6389	probable arabinosyltransferase A
EMB^R MEM/CLA^R II	3157252	snp	C>G	intergenic	-25bp from TSS of MSMEG_3084	glyceraldehyde-3-phosphate dehydrogenase Gap
	5795546	ins	G>GCC	intergenic	-215bp from TSS of MSMEG_5710	hypothetical protein
	6383472	snp	G>A	intergenic	-9bp from TSS of MSMEG_6319	penicillin-binding protein, transpeptidase
	6451445	snp	G920A	Arg307His	MSMEG_6389	probable arabinosyltransferase A
	6861902	snp	T>C	intergenic	-21bp from TSS of MSMEG_6811	conserved hypothetical protein
EMB^R MEM/CLA^R III	3157252	snp	C>G	intergenic	-25bp from TSS of MSMEG_3084	glyceraldehyde-3-phosphate dehydrogenase Gap
	5795546	ins	G>GCC	intergenic	-215bp from TSS of MSMEG_5710	hypothetical protein
	6383472	snp	G>A	intergenic	-9bp from TSS of MSMEG_6319	penicillin-binding protein, transpeptidase
	6451445	snp	G920A	Arg307His	MSMEG_6389	probable arabinosyltransferase A

Table S4. Mutations and affected genes identified on all *Mtb* isolates of this study.

[a] Genome position corresponds to the sequence coordinate on the *Mtb* H37Rv reference genome (GenBank accession number AL123456.3). [b] snp, single nucleotide polymorphism. [c] In the cases where the mutation occurred in a coding region, the nucleotide (Nt) position within the gene is shown. AMX, amoxicillin; CLA, clavulanate; MEM, meropenem.

Isolate	Genome position[a]	Type[b]	Nt change[c]	AA change	Locus tag	Product
AMX/CLA^R I	852106	snp	A499G	Lys167Glu	Rv0757	Possible two component system response transcriptional positive regulator PhoP
	3418847	snp	C530T	Thr177Ile	Rv3058c	Possible transcriptional regulatory protein (probably TetR-family)
	3768564	snp	C30G	Val10Val	Rv3352c	Possible oxidoreductase
AMX/CLA^R II	851902	snp	A295G	Thr99Ala	Rv0757	Possible two component system response transcriptional positive regulator PhoP
	3418847	snp	C530T	Thr177Ile	Rv3058c	Possible transcriptional regulatory protein (probably TetR-family)
	3768564	snp	C30G	Val10Val	Rv3352c	Possible oxidoreductase
AMX/CLA^R III	851902	snp	A295G	Thr99Ala	Rv0757	Possible two component system response transcriptional positive regulator PhoP
	3418847	snp	C530T	Thr177Ile	Rv3058c	Possible transcriptional regulatory protein (probably TetR-family)
	3768564	snp	C30G	Val10Val	Rv3352c	Possible oxidoreductase
MEM/CLA^R I	852161	snp	T554C	Phe185Ser	Rv0757	Possible two component system response transcriptional positive regulator PhoP
	3175952	snp	C1314T	Ala438Ala	Rv2864c	Possible penicillin-binding lipoprotein
MEM/CLA^R II	852161	snp	T554C	Phe185Ser	Rv0757	Possible two component system response transcriptional positive regulator PhoP
	3175952	snp	C1314T	Ala438Ala	Rv2864c	Possible penicillin-binding lipoprotein
MEM/CLA^R III	852161	snp	T554C	Phe185Ser	Rv0757	Possible two component system response transcriptional positive regulator PhoP
	3175952	snp	C1314T	Ala438Ala	Rv2864c	Possible penicillin-binding lipoprotein

Table S5. Information of the European Nucleotide Archive accession numbers of the strains and derivative mutants used in this study.

Species	Strain/isolate name	Alias	BioSample_Id	Run_Id
M. smegmatis	mc^2-155 WT	PT_Msm001	ERS12287434	ERR9882569
M. smegmatis	Δ*blaS*	PT_Msm002	ERS12287435	ERR9882546
M. smegmatis	AMX/CLA^R I	PT_Msm003	ERS12287436	ERR9882547
M. smegmatis	AMX/CLA^R II	PT_Msm004	ERS12287437	ERR9882584
M. smegmatis	AMX/CLA^R III	PT_Msm005	ERS12287438	ERR9882560
M. smegmatis	MEM/CLA^R I	PT_Msm006	ERS12287439	ERR9882555
M. smegmatis	MEM/CLA^R II	PT_Msm007	ERS12287440	ERR9882549
M. smegmatis	MEM/CLA^R III	PT_Msm008	ERS12287441	ERR9882563
M. smegmatis	Δ*blaS* MEM^R I	PT_Msm009	ERS12287442	ERR9882568
M. smegmatis	Δ*blaS* MEM^R II	PT_Msm010	ERS12287443	ERR9882575
M. smegmatis	Δ*blaS* MEM^R III	PT_Msm011	ERS12287444	ERR9882566
M. smegmatis	VAN^R I	PT_Msm012	ERS12287445	ERR9882576
M. smegmatis	VAN^R II	PT_Msm013	ERS12287446	ERR9882558
M. smegmatis	VAN^R III	PT_Msm014	ERS12287447	ERR9882553
M. smegmatis	RIF^R I	PT_Msm015	ERS12287448	ERR9882545
M. smegmatis	RIF^R II	PT_Msm016	ERS12287449	ERR9882561
M. smegmatis	RIF^R III	PT_Msm017	ERS12287450	ERR9882562
M. smegmatis	INH^R I	PT_Msm018	ERS12287451	ERR9882582
M. smegmatis	INH^R II	PT_Msm019	ERS12287452	ERR9882551
M. smegmatis	INH^R III	PT_Msm020	ERS12287453	ERR9882573
M. smegmatis	INH^R AMX/CLA^R I	PT_Msm021	ERS12287454	ERR9882559
M. smegmatis	INH^R AMX/CLA^R II	PT_Msm022	ERS12287455	ERR9882554
M. smegmatis	INH^R AMX/CLA^R III	PT_Msm023	ERS12287456	ERR9882548
M. smegmatis	INH^R MEM/CLA^R I	PT_Msm024	ERS12287457	ERR9882577
M. smegmatis	INH^R MEM/CLA^R II	PT_Msm025	ERS12287458	ERR9882579
M. smegmatis	INH^R MEM/CLA^R III	PT_Msm026	ERS12287459	ERR9882580
M. smegmatis	EMB^R I	PT_Msm027	ERS12287460	ERR9882571
M. smegmatis	EMB^R II	PT_Msm028	ERS12287461	ERR9882585
M. smegmatis	EMB^R III	PT_Msm029	ERS12287462	ERR9882572
M. smegmatis	EMB^R AMX/CLA^R I	PT_Msm030	ERS12287463	ERR9882570
M. smegmatis	EMB^R AMX/CLA^R II	PT_Msm031	ERS12287464	ERR9882550
M. smegmatis	EMB^R AMX/CLA^R III	PT_Msm032	ERS12287465	ERR9882556
M. smegmatis	EMB^R MEM/CLA^R I	PT_Msm033	ERS12287466	ERR9882574
M. smegmatis	EMB^R MEM/CLA^R II	PT_Msm034	ERS12287467	ERR9882581
M. smegmatis	EMB^R MEM/CLA^R III	PT_Msm035	ERS12287468	ERR9882565
M. tuberculosis	H37Rv WT	PT_Mtb001	ERS12287469	ERR9882564
M. tuberculosis	AMX/CLA^R I	PT_Mtb002	ERS12287470	ERR9882544
M. tuberculosis	AMX/CLA^R II	PT_Mtb003	ERS12287471	ERR9882567
M. tuberculosis	AMX/CLA^R III	PT_Mtb004	ERS12287472	ERR9882557
M. tuberculosis	MEM/CLA^R I	PT_Mtb005	ERS12287473	ERR9882583
M. tuberculosis	MEM/CLA^R II	PT_Mtb006	ERS12287474	ERR9882552
M. tuberculosis	MEM/CLA^R III	PT_Mtb007	ERS12287475	ERR9882578
